# Farmland practices are driving bird population decline across Europe

**DOI:** 10.1073/pnas.2216573120

**Published:** 2023-05-15

**Authors:** Stanislas Rigal, Vasilis Dakos, Hany Alonso, Ainārs Auniņš, Zoltán Benkő, Lluís Brotons, Tomasz Chodkiewicz, Przemysław Chylarecki, Elisabetta de Carli, Juan Carlos del Moral, Cristian Domşa, Virginia Escandell, Benoît Fontaine, Ruud Foppen, Richard Gregory, Sarah Harris, Sergi Herrando, Magne Husby, Christina Ieronymidou, Frédéric Jiguet, John Kennedy, Alena Klvaňová, Primož Kmecl, Lechosław Kuczyński, Petras Kurlavičius, John Atle Kålås, Aleksi Lehikoinen, Åke Lindström, Romain Lorrillière, Charlotte Moshøj, Renno Nellis, David Noble, Daniel Palm Eskildsen, Jean-Yves Paquet, Mathieu Pélissié, Clara Pladevall, Danae Portolou, Jiří Reif, Hans Schmid, Benjamin Seaman, Zoltán D. Szabo, Tibor Szép, Guido Tellini Florenzano, Norbert Teufelbauer, Sven Trautmann, Chris van Turnhout, Zdeněk Vermouzek, Thomas Vikstrøm, Petr Voříšek, Anne Weiserbs, Vincent Devictor

**Affiliations:** ^a^Institut des Sciences de l’Évolution de Montpellier (ISEM), Univ. de Montpellier, CNRS, Institut de recherche pour le développement (IRD), École pratique des hautes études (EPHE), Montpellier 34095, France; ^b^Portuguese Society for the Study of Birds Sociedade, Portuguesa para o Estudo das Aves (SPEA), Lisbon 700-031, Portugal; ^c^Faculty of Biology, University of Latvia, Riga LV-1004, Latvia; ^d^Latvian Ornithological Society, Riga LV-1050, Latvia; ^e^Romanian Ornithological Society/BirdLife Romania, Cluj-Napoca 030231, Romania; ^f^Centre de Ciència i Tecnologia Forestal de Catalunya (CTFC), Solsona 25280, Spain; ^g^Centre for Ecological Research and Forestry Applications (CREAF), Cerdanyola del Vallès 08193, Spain; ^h^Spanish National Research Council, Consejo Superior de Investigaciones Científicas (CSIC), Cerdanyola del Vallès 08193, Spain; ^i^Catalan Ornithological Institute, Natural History Museum of Barcelona, Barcelona 4-5 08019, Spain; ^j^Museum and Institute of Zoology, Polish Academy of Sciences, Warszawa 00-679, Poland; ^k^Polish Society for the Protection of Birds, Ogólnopolskie Towarzystwo Ochrony Ptaków (OTOP), Marki 05-270, Poland; ^l^FaunaViva, MITO2000, Parma 43122, Italy; ^m^Spanish Ornithological Society (Sociedad Española de Ornitología/BirdLife), Madrid 28053, Spain; ^n^Patrinat & UMR7204 Centre d'Écologie et des Sciences de la Conservation (CESCO), MNHN: Muséum national d'Histoire naturelle (MNHN)-CNRS-SU, Paris 75005, France; ^o^Sovon Dutch Center for Field Ornithology, Nijmegen 6525, The Netherlands; ^p^Radboud Institute for Biological and Environmental Sciences, Radboud University, Nijmegen 6525, The Netherlands; ^q^The Royal Society for the Protection of Birds (RSPB) Centre for Conservation Science, Sandy SG19 2DL, United Kingdom; ^r^Department of Genetics, Evolution and Environment, Centre for Biodiversity & Environment Research, University College London, London WC1E 6BT, United Kingdom; ^s^British Trust for Ornithology, Thetford IP24 2PU, United Kingdom; ^t^European Bird Census Council, Nijmegen 6524, The Netherlands; ^u^Section of Science, Nord University, Levanger 8049, Norway; ^v^BirdLife Norway, Trondheim 7012, Norway; ^w^BirdLife Cyprus, Nicosia 2340, Cyprus; ^x^BirdWatch Ireland on behalf of the National Parks and Wildlife Service, Kilcoole A63 RW83, Republic of Ireland; ^y^Czech Society for Ornithology/BirdLife Czech Republic, Prague 150 00, Czech Republic; ^z^Društvo za opazovanje in proučevanje ptic Slovenije (DOPPS) BirdLife Slovenia, Ljubljana SI-1000, Slovenia; ^aa^Adam Mickiewicz University, Poznań 61-712, Poland; ^bb^Vytautas Magnus University, Kaunas 44248, Lithuania; ^cc^Lithuanian Ornithological Society (Lietuvos Ornitologų Draugija (LOD)), Vilnius LT-03208, Lithuania; ^dd^Norwegian Institute for Nature Research, Trondheim 7485, Norway; ^ee^Finnish Museum of Natural History, University of Helsinki, Helsinki 00100, Finland; ^ff^Department of Biology, Lund University, Lund 223 62, Sweden; ^gg^Danish Ornithological Society (DOF)/BirdLife Denmark, Copenhagen 1620, Denmark; ^hh^Estonian Ornithological Society/Birdlife Estonia, Tartu 51005, Estonia; ^ii^Aves-Natagora, Namur 5000, Belgium; ^jj^Andorran Research+Innovation, Sant Julià de Lòria AD500, Principality of Andorra; ^kk^Hellenic Ornithological Society, Athens 10437, Greece; ^ll^Institute for Environmental Studies, Faculty of Science, Charles University, Prague 128 00, Czech Republic; ^mm^Department of Zoology, Faculty of Science, Palacký University, Olomouc 779 00, Czech Republic; ^nn^Swiss Ornithological Institute, Sempach CH-6204, Switzerland; ^oo^BirdLife Austria, Vienna 1070, Austria; ^pp^Milvus Group Bird and Nature Protection Association, Tîrgu Mureş 540445, Romania; ^qq^University of Nyíregyháza, Nyíregyháza 4400, Hungary; ^rr^Hungarian Ornithological and Nature Conservation Society (Magyar Madártani és Természetvédelmi Egyesület (MME))/BirdLife Hungary, Budapest 1121, Hungary; ^ss^Dimensione Ricerca Ecologia Ambiente (DREAM) Italia, Pratovecchio 52015, Italy; ^tt^Dachverband Deutscher Avifaunisten, Muenster D-48157, Germany

**Keywords:** anthropogenic pressures, agriculture intensification, bird conservation, large-scale analysis

## Abstract

Declines in European bird populations are reported for decades but the direct effect of major anthropogenic pressures on such declines remains unquantified. Causal relationships between pressures and bird population responses are difficult to identify as pressures interact at different spatial scales and responses vary among species. Here, we uncover direct relationships between population time-series of 170 common bird species, monitored at more than 20,000 sites in 28 European countries, over 37 y, and four widespread anthropogenic pressures: agricultural intensification, change in forest cover, urbanisation and temperature change over the last decades. We quantify the influence of each pressure on population time-series and its importance relative to other pressures, and we identify traits of most affected species. We find that agricultural intensification, in particular pesticides and fertiliser use, is the main pressure for most bird population declines, especially for invertebrate feeders. Responses to changes in forest cover, urbanisation and temperature are more species-specific. Specifically, forest cover is associated with a positive effect and growing urbanisation with a negative effect on population dynamics, while temperature change has an effect on the dynamics of a large number of bird populations, the magnitude and direction of which depend on species' thermal preferences. Our results not only confirm the pervasive and strong effects of anthropogenic pressures on common breeding birds, but quantify the relative strength of these effects stressing the urgent need for transformative changes in the way of inhabiting the world in European countries, if bird populations shall have a chance of recovering.

## Significance

Using the most recent and largest empirical dataset ever assembled for Europe to investigate the effect of anthropogenic pressures, we highlighted the predominant detrimental impact of agriculture intensification on avian biodiversity at a continental scale over climate change, urbanization, and forest cover changes. Our results do not simply quantify correlations, but our analytical design is meant to strive for more quasicausal responses of bird populations to global change drivers. This paper contributes to the highest political and technical challenge faced by agricultural policy in Europe, struggling to balance high productivity from intensive agricultural practices with environmental protection, and the results are therefore crucial to policymakers, scientists, and the general public concerned for biodiversity and global change issues.

Human pressures on biodiversity are intensifying (1), while, at the same time, biodiversity decline is accelerating. Global reductions have been reported in a wide variety of groups, including common species (2), and ranging from marine and terrestrial vertebrates to insects (3–5).

Birds are the largest group of terrestrial vertebrates in species number and are widely affected by ongoing global change (6, 7). Bird populations have been monitored for decades in many countries, and their traits (e.g., thermal preference, diet, habitat specialization) are well documented (8, 9). Major population trends highlighting declines in abundance and diversity have been reported both in specific countries (10, 11) and at continental scale, e.g., in Europe (12, 13) and North America (14). Beyond such global approaches, trend analyses related to species traits (15, 16) have highlighted which species have been the most impacted and suggest that some categories of species are more affected than others. For instance, the widespread decline of species in farmland or grassland habitat is particularly well documented (10), as well as the less pronounced decline in woodland species (17). Other species’ ecological traits such as thermal preference, habitat specialization, synanthropy [the tendency to positively select human-dominated habitats (18)], as well as migratory strategies have been claimed to explain, to some extent, large-scale and long-term bird population dynamics (19, 20).

These differences in the response of species grouped as a function of a shared criterion (e.g., the main type of habitat) have been useful to point out anthropogenic pressures driving avian biodiversity decline. Land-use change, agriculture, biodiversity resource exploitation, and climate change are among the main threats (7, 20–22). More precisely, analyses conducted at local to continental scales pointed out the role of agricultural intensification [i.e., changes in farming practices leading to an increase of chemical inputs and a reduction of habitat heterogeneity (23)] in explaining the decline of farmland birds (24, 25), while land-use modification such as change in forest cover or urban sprawl and climate change were important pressures for other groups of species (21). Yet, the relative effects of multiple pressures on population dynamics have hardly been tested at large spatial scale as bird populations’ responses to those pressures remain mostly assessed using a limited set of pressures at a continental scale (26). Moreover, the current knowledge on bird populations’ responses to those pressures is mostly based on indirect correlative approaches, thus limiting the scope of interpretation (27, 28). In this context, we still lack understanding of how these major anthropogenic pressures affect large-scale spatiotemporal dynamics of European bird populations.

To extend and complete the findings of the previous large-scale studies (26, 29), we propose an approach that aims at 1) ranking pressures according to their overall effect on bird population dynamics and 2) strengthening the existing correlational results on the relationship between pressures, bird species, and their ecological traits. We therefore conduct an original combined analysis based on *trends* [using a partial least square regression (PLS) (30, 31) to estimate the overall effect of each pressure on European common birds as well as the relative strength of these effects] and *time series* (to verge on causal links between pressures and responses) of bird population and pressures. Several methods can help striving for causal links between drivers and species responses, notably using evidence accumulation or the removal of confounding factors (27). Another option is to use time series analysis. Recent methods such as convergent cross mapping (CCM) and S-map (32–34) have made it possible to detect and quantify “causal” relationships between time series for species and anthropogenic drivers and pressures. As such, a causality has been defined in the context of dynamical systems (35) and does not emerge from experimental design (28); we will refer to it as *quasicausality* to avoid misinterpretations of the concept. CCM and S-map are based on state space reconstruction from time series and can be used: i) to determine links between time series of abiotic factors and species time series (36, 37) and ii) to quantify the strength of such influences (38). This approach therefore provides a means of assessing the influences of specific drivers that is complementary to trend analysis, as influences are estimated for each species and can then be related to species traits.

In the present study, we assess the effects of four major anthropogenic pressures, including agricultural intensification measured as the cover of farms with high input of pesticides and fertilizers, land use with change in forest cover, urbanization, and climate change measured as temperature change, on the large-scale spatiotemporal dynamics of European bird populations. We use the largest dataset available (39) resulting from the combination of standardized Breeding Bird Surveys conducted in 28 countries from 1980 to 2016 (representing 170 common bird species monitored at more than 20,000 sites with standardized protocols) to: 1) state the large-scale spatiotemporal dynamics of European bird populations over 37 years; 2) relate them to global spatiotemporal dynamics of the four pressures over the last decades, based on the analyses of trends and time series; and 3) investigate whether and which combinations of species traits were more prone to be positively or negatively affected.

## Results

### 1) Bird Population and Pressure Dynamics.

**Fig. 1.**
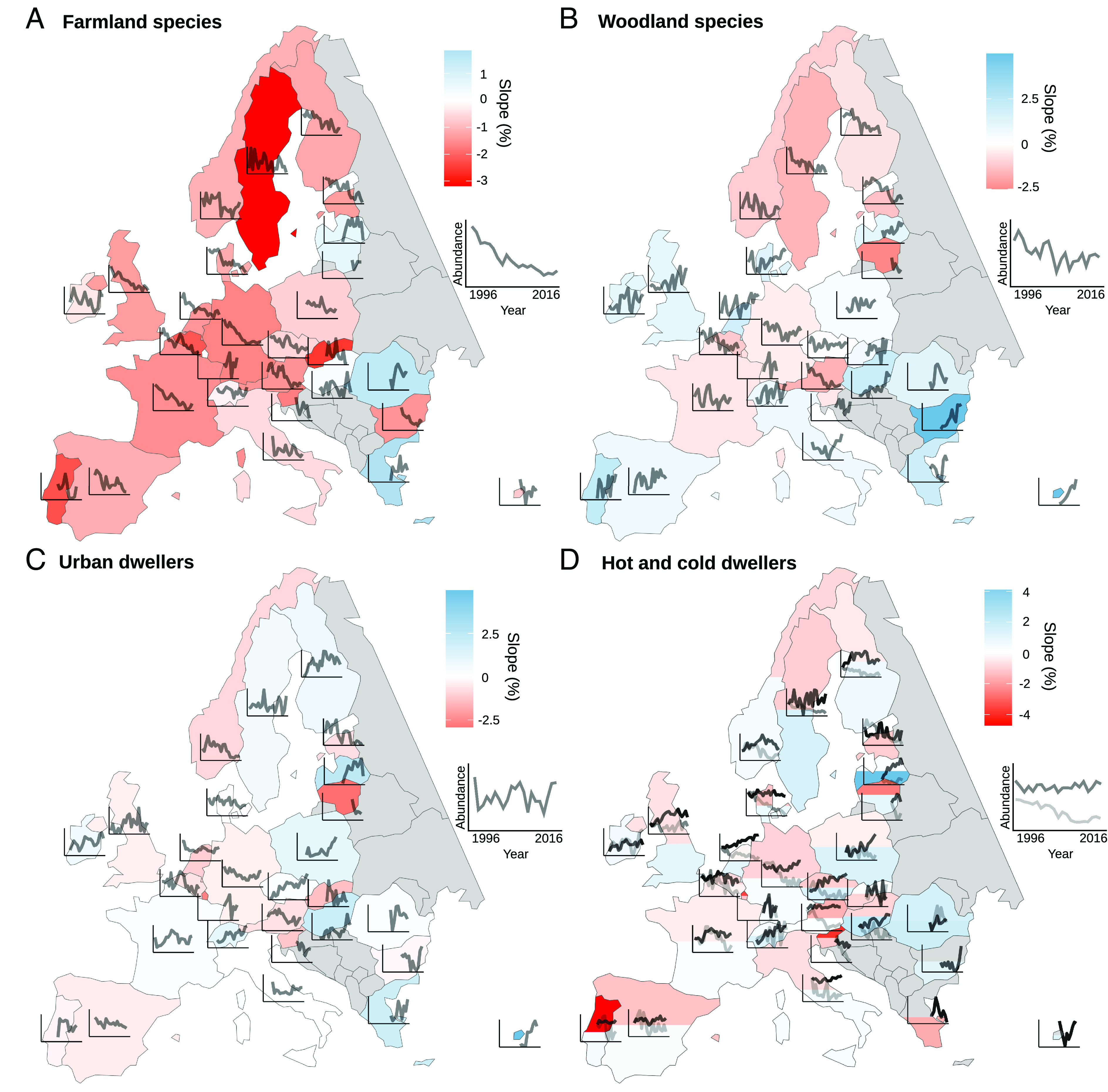
Temporal change in bird abundance in Europe between 1996 and 2016 for countries participating in the PanEuropean Common Bird Monitoring Scheme (PECBMS) (n = 28, non-PECBMS countries in gray). For each country, the color represents the slope (red for decline, blue for increase) and the black line corresponds to the time series of the multispecies index (MSI) between 1996 and 2016 (species lists by country in *SI Appendix*, *Appendix 5*). (*A*) Change in abundance of farmland species (MSI by country on 19 species) showing an overall sharp while decelerating decline. (*B*) Change in abundance of woodland species (MSI by country on 25 species) showing an overall linear decline. (*C*) Change in abundance of urban dwellers (MSI by country on 22 species) showing an overall stable trajectory. (*D*) Change in abundance of cold dwellers (light gray, MSI by country on 35 species) showing an overall linear decline. Change in abundance of hot dwellers (dark gray, MSI by country on 35 species) showing an overall stable trajectory. Color for hot dweller trends on the southern part of countries and color for cold dwellers on the northern part of countries.

Common bird time series in Europe have shown a general decline in abundance between 1980 and 2016 (−25.4% ± 2.8) (Fig. 1 and see trends for each period in *SI Appendix*, *Appendix 1*). This decline is not equally distributed among the different groups of species. Specifically, farmland species populations have been more affected (−56.8% ± 4.9, Fig. 1*A*) than other groups of common birds such as woodland birds (−17.7% ± 9.0, Fig. 1*B*), urban dwellers (−27.8% ± 3.6, Fig. 1*C*), cold dwellers (−39.7% ± 3.1, Fig. 1*D*), and hot dwellers (−17.1% ± 8.1, Fig. 1*D*). Moreover, farmland and cold dweller species have been universally declining in almost all European countries (Fig. 1 *A* and *D*), except for some Eastern countries for which monitoring data are available over a shorter period of time, while trends are more diverse between countries for woodland and urban dwellers (Fig. 1 *B* and *C*).

**Fig. 2.**
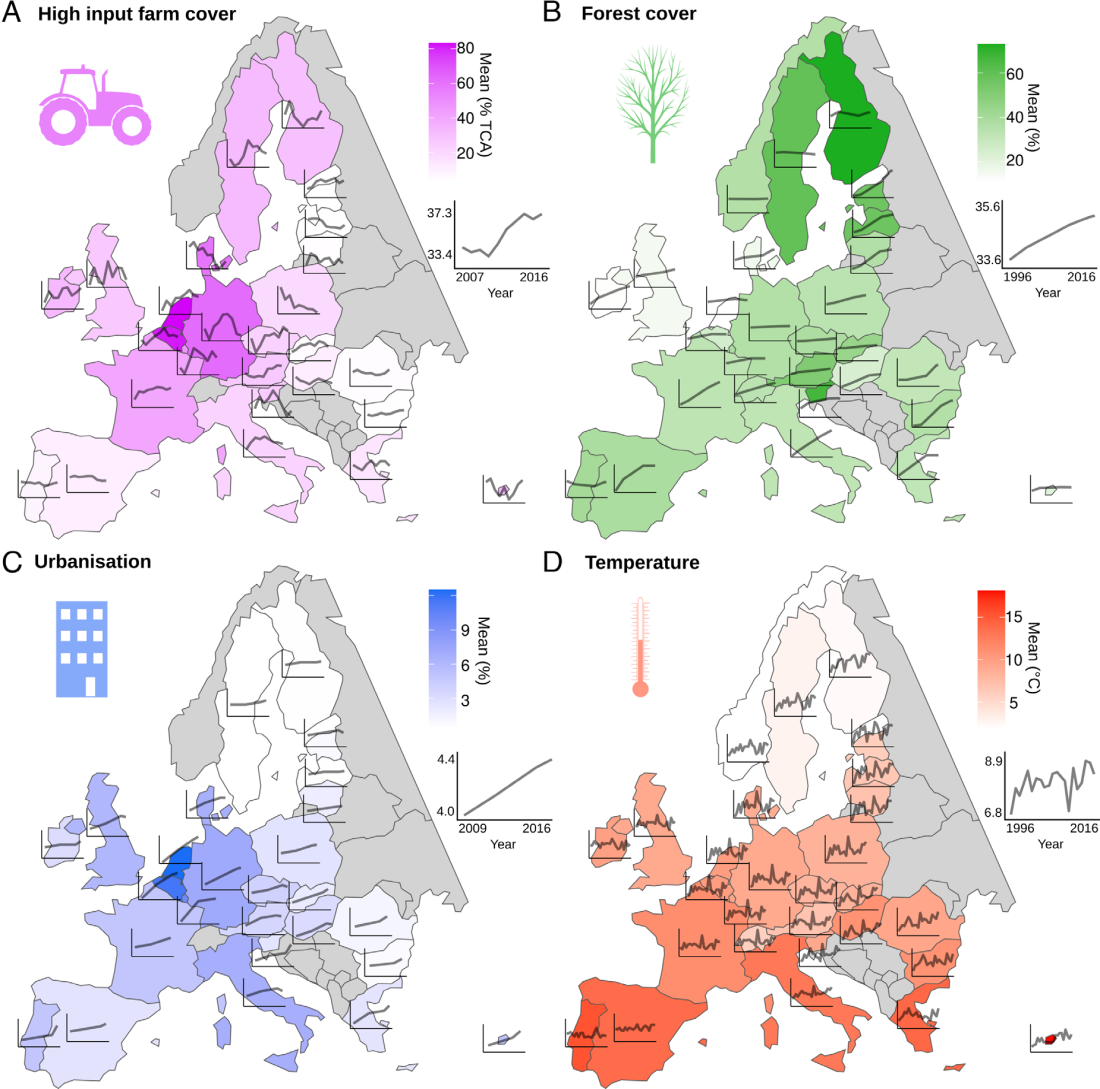
Anthropogenic pressures for countries participating in the PanEuropean Common Bird Monitoring Scheme (PECBMS) (n = 28, non-PECBMS countries and countries with no available data in gray). For each country and each pressure, the color represents the mean and the black line corresponds to the time series. (*A*) High-input farm cover (% of total cultivated area covered by high-input farms), period covered by data 2007 to 2016. (*B*) Forest cover (% of the country’s surface), 1996 to 2016. (*C*) Urbanization (% of the country’s surface), 2009 to 2016. (*D*) Temperature (°C), 1996 to 2016.

Significant heterogeneity in potential drivers of bird population changes exists among countries, notably with respect to the type and intensity of land-use changes (Fig. 2). For instance, agricultural intensification (+2.1% ± 0.9 between 2007 and 2016, Fig. 2*A*) and urbanization (+0.4% ± 0.0 between 2009 and 2016, Fig. 2*C*) are more severe in western compared to eastern European countries. Temperature change is faster at high latitudes (+13.2% ± 10.5 between 1996 and 2016, Fig. 2*D*), while the progression of natural forests or forest plantations is country dependent (+2.1% ± 0.1 between 1996 and 2016, Fig. 2*B*).

### 2) Effects of Pressures on Bird Populations.

**Fig. 3.**
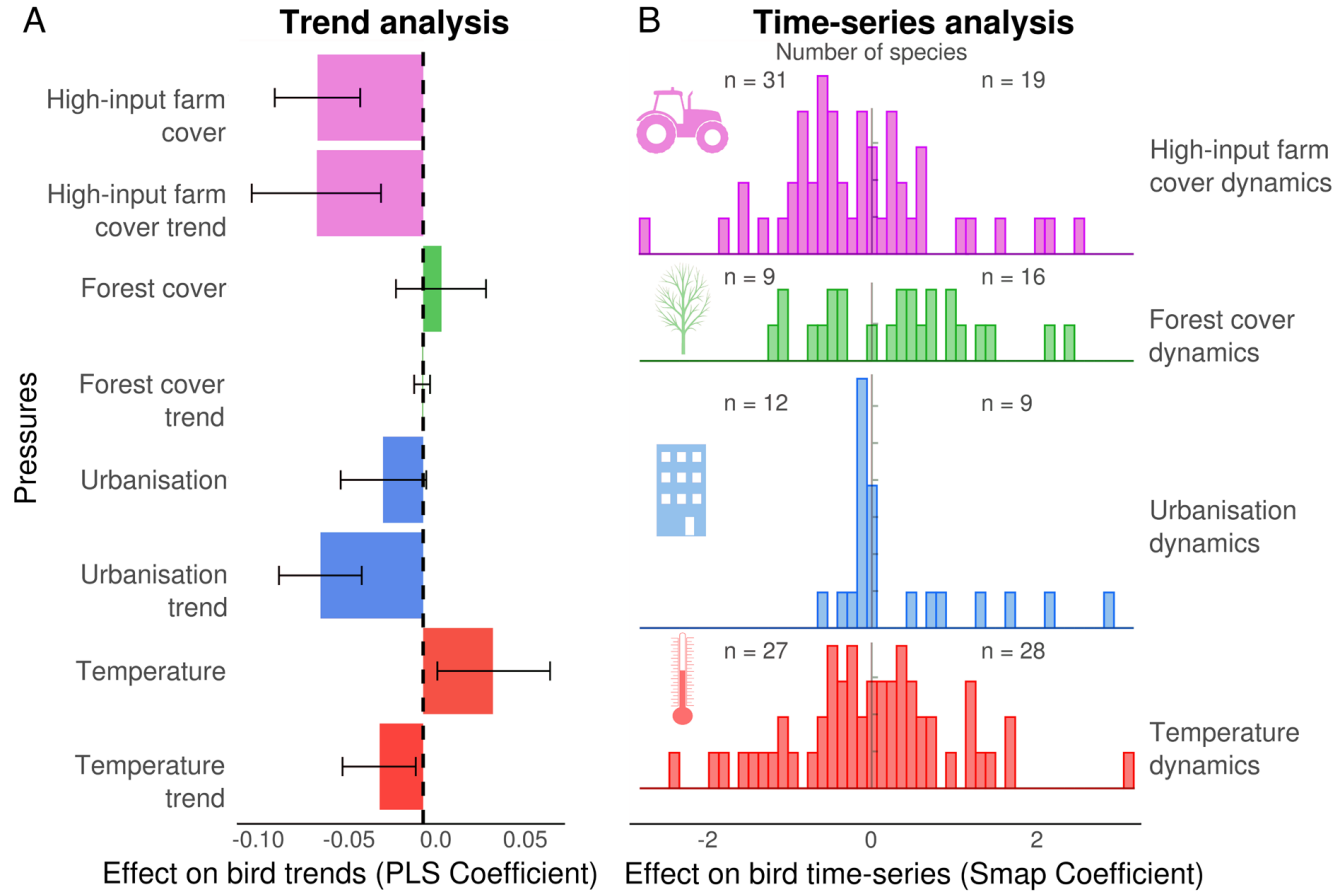
Relationship between anthropogenic pressures and bird trends and time series. (*A*) Relative effects of high-input farm cover, forest cover, urbanization, and temperature and their trends on bird trends (1996 to 2016, 141 species) obtained by partial least square regression (PLS). Bias-corrected and accelerated CIs are displayed. (*B*) Distribution of the strength of the influence of pressures (scaled S-map coefficients) on bird time series. The number of species with negative and positive mean S-map coefficients is shown.

The trend analysis (PLS) reveals that agricultural intensification is the main pressure negatively related to species trend (PLS coefficient for high-input farm cover = −0.037 ± 0.015, PLS coefficient for high-input farm cover trend = −0.037 ± 0.022, Fig. 3*A* and supplementary results in *SI Appendix*, *Appendix 2*). Growing urbanization cover is also negatively related to species trend (PLS coefficient for urbanization trend = −0.036 ± 0.015, Fig. 3*A*). Forest cover change is not related to an overall positive or negative change in common birds (PLS coefficient for forest cover = 0.000 ± 0.003, Fig. 3*A*). Temperature change is negatively related to species trends (PLS coefficient for temperature trend = −0.015 ± 0.013, Fig. 3*A*).

Using CCM and S-map, we found that most species are negatively affected by high-input farm cover (31 of the 50 species for which an impact was identified, Fig. 3*B*). This analysis also confirms the negative influence of urbanization, related to 21 species time series (12 negatively and nine positively, Fig. 3*B*). In contrast, forest time series have been more positively related to species time series (nine negatively and 16 positively of the 25 for which an impact was identified, Fig. 3*B*). Finally, temperature time series effects were balanced between the 55 species time series significantly impacted (27 negatively and 28 positively, Fig. 3*B*).

### 3) Trait Syndrome.

**Fig. 4.**
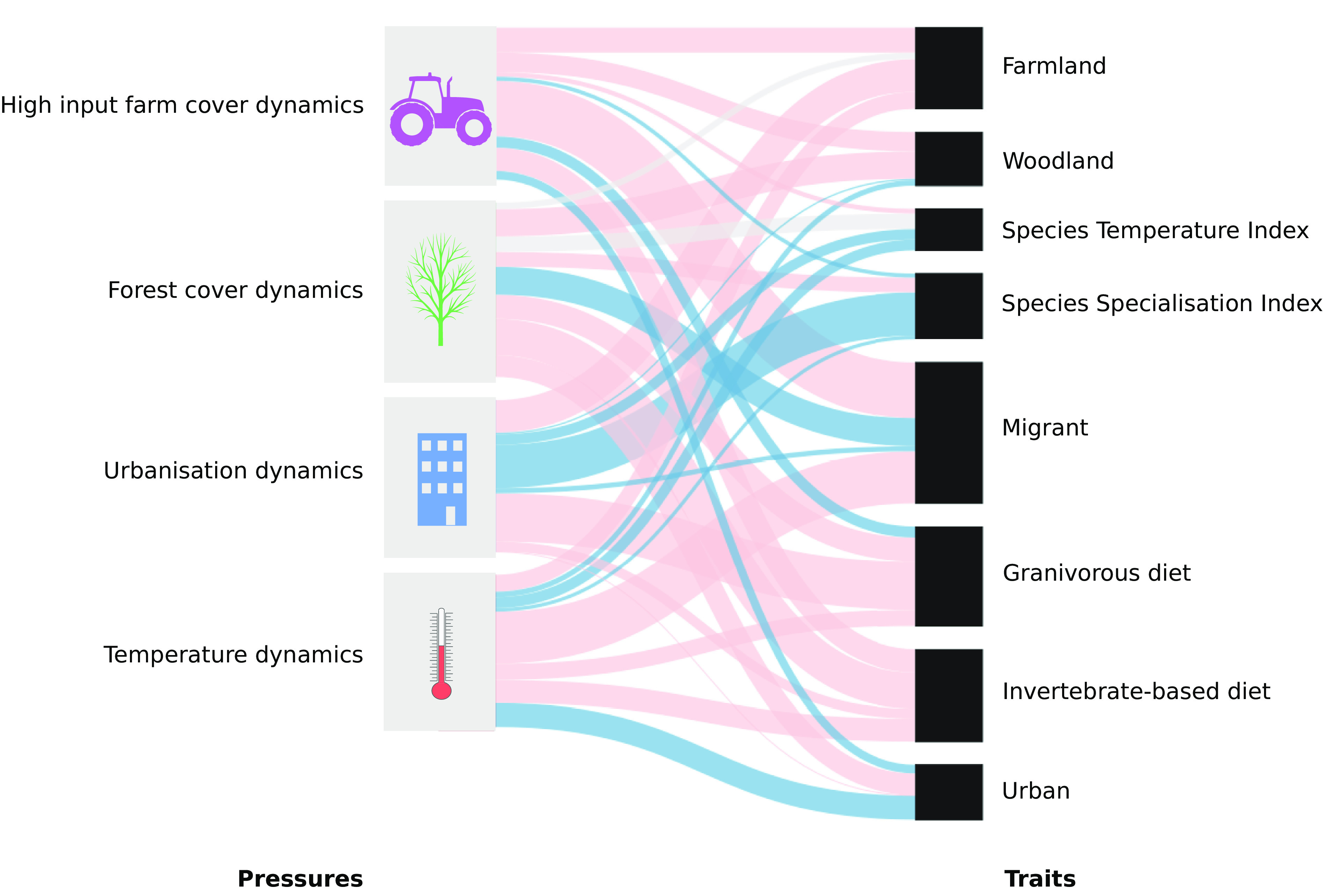
Results of the partial least square regression between each pressure influence on species time series and species traits. Nonsignificant effects are shown in gray, negative effects are shown in light red, and positive effects are shown in blue. The magnitude of the effect is displayed by the line width, scaled for each pressure.

It is worth noting that for every pressure, some species may still benefit from it, while many others are negatively affected. We therefore analyze the specific ecological traits (8) shared among species impacted by pressures (Fig. 4). We find a mostly negative influence of high-input farm cover not only for farmland species, but also for species with a diet at least partly based on invertebrates during the breeding season, long-distance migrants, and woodland birds, i.e., a vast majority of the common birds (8). Forest cover mostly positively influenced long-distance migrants. Farmland species, granivorous species, and species with an invertebrate-based diet have been mostly negatively impacted by urbanization. Finally, the influence of temperature has been mostly positive for hot dwellers, urban dwellers, woodland species, and specialists, but mainly negative for cold dwellers, long-distance migrants, farmland species, generalists, and species with an invertebrate-based or granivorous diet.

## Discussion

To our knowledge, our study provides one of the most complete analyses, at the continental European scale, of the effect of anthropogenic pressures on common breeding bird population dynamics. While previous studies have represented an essential step forward for the understanding of bird population decline at large scale (20, 26, 29), our study provides two critical developments by measuring the relative importance of four main pressures at large scale and with quasicausal estimates.

At the continental European scale, the negative relationship between pesticide and fertilizer use corresponds to the main driver of the decline of bird populations. Until now, habitat preference of a species has been a key factor in assessing the impact of anthropogenic pressures (29). In particular, the sharp decline of farmland birds has been more and more related to agricultural intensification, and especially pesticide use, in Europe and North America (27, 40). Here, both the PLS analysis of the relative effect of major anthropogenic pressures and the CCM quasicausal approach point out that high-input farming is the most influencing pressure explaining bird population changes, and not only for farmland species. This negative effect is also visible in countries with lower average agricultural intensity, as the effect of an intensification is even greater in these countries (see interacting effects in *SI Appendix*, *Appendix 2*). Furthermore, bird populations in countries with smaller agricultural production units are in better condition (*SI Appendix, Appendix 2*), indicating that the increase in the size of production units, another key aspect of agricultural intensification, also contributes to the decline of bird populations, probably through the reduction of habitat heterogeneity (41). We admit that the data on the use of chemical inputs (pesticides and fertilizers) are still very coarse and this does not allow us, for example, to understand the complex mechanisms behind the relationship we uncover. Given the crucial importance of this pressure, legislation on the availability of such data on input use at a precise spatial and temporal scale for all European countries should be strengthened. However, many of the deleterious impacts of agricultural intensification are known, specifically those of pesticides and fertilizers on insects and other invertebrates that may imply trophic cascade effects on birds (42). Invertebrates represent an important part of the diet for many birds in at least some development stages. They are particularly crucial during the breeding period for 143 species among the 170 studied species for which, for instance, a reduction in food availability is likely to impact reproductive success by modifying parental behavior and nestling survival in addition to direct contamination by seed consumption and trophic accumulation with sublethal effect (43).

Beyond farmland practices, other drivers are at play. Urbanization, which has increased in all European countries, can also be related to the overall decline of the avifauna. Although a detailed analysis of the specific link between urbanization and each species might require more accurate time series for urbanization, the available data suggest a negative impact for most species. Forest cover dynamics tend to be mostly related to an increase in bird populations, but the overall increase in forest cover is not reflected by the dynamics of woodland species populations, nor is it visible in the trends of the entire pool of species. Forest cover increase in Europe over the last decades can hide other changes, notably in forest quality, such as a decline of old-growth forests that are essential to many woodland resident species (44). More widely, different impacts are expected on birds between reforestation by managed forests and regrowth after agricultural abandonment, and the subsequent closure of habitats is not likely to benefit open habitat species.

Finally, temperature change not only has had an overall negative impact on the common avifauna as a whole, but also clearly segregated positively affected species, mainly hot dwellers, from negatively affected species, mostly cold dwellers. The effect of temperature change must therefore be considered in parallel to the ability of species to track change in temperature both in space and time. The climatic debt of birds’ response to climate change, caused by the time lag in their geographic range shift, is amplified by the gap between this group and the response of other species with which they directly interact (45). This is particularly challenging for migratory species, with long-distance migrants more strongly and negatively affected by temperature than short-distance migrants and residents (20). The shift in resource availability and optimal environmental conditions caused by climate change has already been documented for specific subsets of the species, period, and countries considered in our study. For instance, migratory forest species have been shown to lag behind the peak of resources during the breeding season, probably due to the change in resource phenology (46). Other studies have emphasized the gradual change in local community composition related to the relative success of hot dwelling species (47, 48). Our results suggest that climate change (temperature) has been a dominant factor in bird population dynamics at a continental scale in recent decades. We also confirm that this effect is even more pronounced in high-latitude (colder) countries (see interacting effects in *SI Appendix, Appendix 2*) (49, 50). Overall, while temperature change can lead to an increase in the distribution and abundance of some species, for those (especially cold dwellers) already affected by other anthropogenic pressures, temperature change constitutes a double burden acting directly on their annual cycle (51).

The tremendous negative impact of agricultural intensification on birds has long been reported in particular for farmland and insectivorous birds, but our study provides strong evidence of a direct and predominant effect of farmland practices at large continental scales. Considering both the overwhelming negative impact of agricultural intensification and the homogenization introduced by temperature and land-use changes, our results suggest that the fate of common European bird populations depends on the rapid implementation of transformative change in European societies, and especially in agricultural reform (52).

## Materials and Methods

### Pressure Baselines and Trends.

Agricultural intensification and forest cover data were obtained from the statistical office of the European Union (Eurostat) (53). The surface covered by high-input farms (as a percentage of the country surface area) for each year between 2007 and 2016 was taken as a proxy for input use. Input values correspond to per-hectare spending on inputs (pesticides and fertilizers). This proxy is used by the European Environmental Agency to estimate the cover of high-input farms, defined as farms where the input value was above the 66th quantile of the distribution [560€.ha^−1^ in 2020 (53)]. Note that high-input farm cover is not correlated with farm size but is highly correlated with pesticide sales and fertilizer consumption (*SI Appendix*, *Appendix 3*). We chose this measure as it can account for price differences among products and countries and can discriminate between farm types (53). The percentage of forest cover was calculated as the number of hectares covered by forest and other wooded land [from the FAO (54)], divided by the country’s surface area, between 1996 and 2016. We used data from Eurostat (55) to assess urbanization values for each country in 2009, 2012, 2015, and 2018. These values are obtained from Land Use and Cover Area frame Survey and correspond to sealed areas, i.e., roofed builtup areas, artificial nonbuiltup areas, and other artificial areas. To obtain annual values between 2009 and 2016, data have been linearly interpolated following the protocol used by the FAO for land-use data (54). Temperature data were extracted from the E-OBS v20.0e database (56) which contains daily mean temperature between 1950 and 2018 over a regular grid of Europe at a scale of 0.1 degree (ca. 111 × 70 km^2^ at European latitudes). For each grid cell, daily temperatures were averaged between August from the year preceding the monitoring and June of the monitoring year as both breeding and nonbreeding season temperatures could have an effect on birds (57). Averaging across cells within a country yielded an annual mean temperature for each country, for each year from 1996 to 2016. We chose to use a homogenized dataset for each pressure (i.e., not coalescing data from different sources for a given pressure). This implied that no high-input farm cover and urbanization data were available for Norway and Switzerland. At country scale, trends in high-input farm cover, forest cover, urbanization, and temperature corresponded to the relative change calculated as the slope of a linear regression between annual pressure values (the response variable), and years (the explanatory variable) scaled by the first pressure value.

### Bird Data.

#### Species time series and population abundance.

Annual Species Abundance Indices (SAI) for birds came from national breeding bird surveys coordinated by the PanEuropean Common Bird Monitoring Scheme (PECBMS) (39) (see examples in *SI Appendix*, *Appendix 4*). Skilled volunteers in each country use comparable standardized protocols (point counts, line transects, and territory mapping) and conduct annual counts at given sites. Overall, the data that are collated in this paper represent the monitoring of more than 20,000 sites, which is one of the largest databases of its kind ever analyzed (see also refs. 6 and 58). TRends and Indices for Monitoring (TRIM) software was used to produce national population indices from site counts, taking into account error estimates and missing observations (59, 60). The dataset initially contained national indices for 170 bird species in 28 countries: 25 from the European Union (excluding Croatia and Malta), the United Kingdom, Norway, and Switzerland (see *SI Appendix*, *Appendix 5* for a list of species and the number of species by country).

In addition to the SAI, which corresponds to a relative value of annual population size, national estimates of the number of breeding pairs were manually extracted from Birdlife Data Zone which collates data from the EU Bird Directive Reporting, to evaluate annual absolute abundance (national population estimate) (13) (see methods for species abundance in *SI Appendix*, *Appendix 1*).

Supranational analyses (i.e., trend analyses at the European scale) of change in abundance were based on species-monitoring data that were available from 1980. We focused on the period 1980 to 2016 to maximize spatial and temporal coverage, with limited uncertainty. The longest time series covered the period 1966 to 2016, but most schemes began only in the 1990s and before 1980, data were only available for six countries in northern Europe. These criteria resulted in a dataset of 1,983 time series, for 115 bird species (among the 170), in the 28 countries over 37 years. Supranational indices (i.e., species indices at the European level) were obtained using the *rtrim R* package (59, 61) with national population size used for weighting. Abundance indices for the whole species pool, or specific (e.g., main habitat) subgroups, were obtained from supranational indices using a multispecies index (MSI) reconstruction (62), adapted for abundance data (see *SI Appendix*, *Appendix 1* for relative abundance MSI and biomass MSI).

National analyses were based on the best trade-off between spatial and temporal cover data. We therefore selected time series beginning in 1996 (±1 y) and ending in 2016. This resulted in a dataset of 1,041 time series, for 141 species (83% of the 170 species), in 14 countries, covering 60% of the area of the 28 countries (see *SI Appendix*, *Appendix 6* for the influence of the choice of period on data distribution).

Both supranational and national trajectories were assessed using a method incorporating data uncertainty and testing for nonlinear trends (63).

#### Bird habitat and ecological traits.

We followed the latest PECBMS classification (https://pecbms.info/) to classify farmland and forest species according to their predominant habitat. Species affinity for urban areas was obtained from the European Nature Information System (EUNIS) database (https://eunis.eea.europa.eu) (64). The Bird EUNIS database provides information on the associations between bird species and habitat types grouped by Mapping and Assessment of Ecosystems and their Services ecosystems (12 types: coastal, cropland, grassland, heathland/shrub, marine/inlets, ocean, rivers/lakes, shelf, sparsely vegetated, urban, wetland, and woodland/forests). All species that have been reported to breed, even partially, in urban areas are considered as urban species. This study covers a continental scale from the Mediterranean to the arctic parts of Europe. Within such large areas, bird species may vary in habitat affiliation. So, national statistics related to birds and biodiversity may include different sets of species than those used here, meaning that the statistics here presented for individual countries may differ from the countries’ own national statistics. Habitat specialization was assessed using the species specialization index (SSI) calculated for the European avifauna (64). For each species, the species temperature index (STI) represents the thermal centroid of its European distribution range (45). STI values have proven useful in predicting the sensitivity of hot *vs*. cold dwellers to climate change (65) (for the detailed list of species and their habitats, see *SI Appendix*, *Appendix 5*). Data regarding migratory strategies (long-distance migrant: beyond Western Palaearctic in nonbreeding season; others: sedentary, facultative, or short-distance migrant) and breeding-season diet (two main classes, granivorous: over 10% of seeds and no other diet types over 10%; invertebrate based: over 10% of arthropods and over 10% of other invertebrates (e.g., molluscs and annelids) and no other diet types over 10%) were extracted from a comprehensive dataset of traits for all European breeding birds (8).

### Statistical Analyses.

#### Trend analysis.

At the European scale, the overall effects of anthropogenic pressures and drivers were identified using a PLS regression (Fig. 1*A*). This approach is a multivariate analytical model that can handle numerous explanatory variables and multicollinearity (30, 31). The PLS method is a combination of multiple regression and principal component analysis, in which several (potentially related) explanatory variables are used to build latent factors (i.e., linear combinations of variables that maximize the explained variance of a response variable). The effects of the explanatory variables on the response variable are estimated as the relationship between these explanatory variables and latent factors. Crossvalidation is used to select the number of components, and this leads to a distribution of components from which an empirical measure of significance for the effect of each explanatory variable can be obtained (66). The PLS coefficient of each explanatory variable corresponds to the effect average across each of the selected components.

In this study, the response variable was the national species trend between 1996 and 2016, and the explanatory variables encompassed four anthropogenic pressures, both in terms of baseline conditions and temporal changes: high-input farm cover and its temporal trend, forest cover and its temporal trend, urbanization and its temporal trend, and finally temperature and its temporal trend. The PLS analysis was performed using the *plsRglm* R package (67), which can handle incomplete data. It also provides significance levels for explanatory variables through bootstrapping and can compute bias-corrected and accelerated CIs (BC_a_) (68) that correct for skewness and bias of the bootstrap distribution.

#### Time series analysis.

Results from the trend analysis help to assess the relative importance of each pressure but are based on correlations and at the scale of the entire pool of species. We therefore attempted to identify direct links between the population dynamics of each bird species, in addition to the analysis of the overall effect of pressures on species trends, by estimating the influence of each pressure time series (high-input farm cover, forest cover, urbanization, temperature) on each species time series (Fig. 1*B*) using two tools of empirical dynamic modeling based on state-space reconstruction: cross convergent mapping (CCM) and S-map (32, 69). In short, significant relationships between pressure and species time series are identified by CCM, a method designed to identify “causality” [as defined in dynamical systems (35)] among time series, and then quantified using the sequentially locally weighted global linear maps [S-map (69)]. The time series analysis is therefore complementary to the trend analysis and uses all available temporal information and tests the effect of each pressure on each species. Detailed tests of the method for robustness to data removal and sensitivity to interaction strength between pressures and species are available in *SI Appendix*, *Appendix 7*.

##### CCM.

In this study, we used the multispatial CCM (33) which is an extension to CCM that can handle short time series (the classical CCM requires series of over 30 time steps) and has been used at continental scale (37). For each of the 170 species, and each of the 28 countries, we combined species time series (national indices) and pressure time series over the same period (2007 to 2016). We then reconstructed pseudo-time series by aligning time series from the different countries for a given species.

More specifically, CCM detects whether the time series of pressure X can be predicted from the time series of species Y by exploring whether M_X_, the attractor manifold of X (defined as the set of states reconstructed from the original and lagged time series of X), can be estimated from M_Y_, the attractor of Y. In that case, the pressure X has a signature in the time series of species Y, *i.e.,* the pressure X is affecting species Y. The implementation of CCM is performed in three steps:

–Estimating the appropriate embedding dimension E to reconstruct attractor manifolds of X and Y. According to Taken’s theorem (70), the core of the CCM method, it is possible to reconstruct *M_X_* using several time lags of the time series of X. Hence, the first of the three steps is to find the best embedding dimension *E* to precisely map the original manifold *M*. In multispatial CCM, time series are reconstructed from several sites, and *E* can thus not be higher than *m,* the minimum number of time steps by site (*E* ≤ m*-1* as one time step must be kept for prediction).
–Testing for nonlinearity in time series to remove stochastic processes. Once the best *E* is determined, one needs to check whether dynamics are strongly influenced by noise, leading to a purely random system, or not. To do so, a part of the observations is used to make predictions for future and increasingly distant observations, and their predictive power is estimated. If the system is nonlinear and not driven by an important stochastic noise, the predictive power should decrease with temporal distance.
Applying the CCM algorithm once E is determined and nonlinearity verified.

CCM results in a crossmap skill coefficient *ρ* which indicates whether time series are quasicausally related [as such causality is defined in the context of dynamical systems (35) and does not emerge from experimental design (28)]. As the information of the entire dynamical system is incorporated in any time series of this system, the influence of other covariates is implicitly taken into account. In multispatial CCM, a bootstrap routine is used to estimate *ρ*. Once CCM has distinguished pairs of causally related time series and the direction of the relationship (X -> Y, Y -> X, or X <-> Y), the S-map method can quantify it (69).

##### S-Map.

In the S-map method, an attractor manifold can be reconstructed by projecting causally related time series in a state space. That is, a manifold represents the ensemble of the system states described by a set of causally related time series. In reverse, the attractor manifold describes how the time series are related in time. By definition, a manifold is a locally Euclidean n-dimensional topological space. That is, in the close neighborhood of an attractor state, the relation between variables is linear and is defined as the partial derivatives between variables. S-maps therefore correspond to a locally weighted multivariate linear regression that empirically and sequentially estimates the Jacobian elements of a variable *x*(*t*) in the state space.

Let $x\left(t\right)=\left\{x_{1}\left(t\right),x_{2}\left(t\right),\ldots ,x_{E}\left(t\right)\right\}$ be the spatial state reconstruction at a given time *t* of a system with *E* interacting species, i.e., the position of the attractor at t. Let t* be a target time. Then, S-maps aim at producing the best local linear model *C* predicting the future value *x*_i_(t*+p) from *x*(t*) as follows:

$${\hat{x}}_{i}\left(t^{*}+p\right)=C_{0}+\sum_{j=1}^{E}C_{ij}X_{j}\left(t^{*}\right).$$

Each local regression is fitted to all vectors of the state space but weighted so that the closer the observation points *x*(*t*_k_) are from the target attractor state *x*(t*), the weightier they are. For given observation *k*, the weight is defined as follows:

$w_{k}=\frac{e^{-\theta ∥x\left(t_{k}\right)-x\left(t^{*}\right)∥}}{\bar{d}}$ with $\bar{d}=\frac{\text{1}}{n}\sum_{j=\text{1}}^{n}\;∥\;X\left(t_{j}\right)-X\left(t^{*}\right)\;∥$ the average distance, *θ* the nonlinear parameter tuning the amplitude of the *C* coefficients. We tested values of *θ* between 0 and 10 and the best *θ* value resulted from a trade-off between a contraction of the coefficient variability and overemphasizing the points closest to *x*(t*).

*C* is therefore a solution to the singularity value decomposition of B=A.C where *A* is the *n x E* dimensional (with *n* the number of observations) matrix of the weighted state space vectors, $A_{kj}=W_{k}X_{j}\left(t_{k}\right)$ , and *B* is the *n* dimensional vector of the future values of *x_i_*, $B_{k}=W_{k}X_{i}\left(t_{k}+p\right)$ . The Jacobian elements are defined as partial derivatives in the multivariate state space and thus, they can be approximate using the coefficient of the weighted linear model *C*. The temporal average value of the S-map coefficients can then be used to quantify the effect between the causally related time series (34, 38).

The number of embedded causal time series should be the same as the best embedding dimension E obtained in the first step of the CCM implementation (38). In addition to the time series of the species Y, we selected *E-1* pressure time series found as causally related to the species time series by CCM. If the number of pressure time series causally related to the species time series was higher than *E-1*, we selected the first *E-1* pressure time series based on the significance of their crossmap skill coefficient *ρ.* The quantitative effect of high-input farm cover, forest cover, urbanization, and temperature change has therefore been estimated simultaneously. We computed S-map between pressures and species time series for each country and used the average S-map coefficient of each pressure on each species across countries as an estimate of the effect of the given pressure on the given species.

#### Trait analysis.

Finally, we investigated which traits were linked to each pressure using a PLS regression. Here, the interaction value (i.e., the effect of pressure time series on species time series) was the response variable and traits were explanatory variables. Discrete explanatory variables are species habitat (farmland, woodland, urban), migratory strategies [long-distance migrants, others (short-distance migrants, facultative migrants, and residents)], and diet (granivorous, invertebrate based). Continuous explanatory variables are SSI and STI. Multicollinearity exists among these traits (*SI Appendix*, *Appendix 8*), but PLS is specifically designed to handle correlated explanatory variables (66).

## Supplementary Material

Appendix 01 (PDF)

## Data Availability

All analyses were conducted using R software (version 3.4.4). Data are already available (39) and the R script is available on Github https://github.com/StanislasRigal/Drivers_European_bird_decline_public (71).
