# Supplementary material for: Farmland practices are driving bird population decline across Europe
Source: Proc Natl Acad Sci U S A. 2023 May 15;120(21):e2216573120. doi: 10.1073/pnas.2216573120 (PMC10214186; doi:10.1073/pnas.2216573120)
Supplement: Supplementary file 1 — Appendix 01 (PDF) [file pnas.2216573120.sapp.pdf]

PNAS

Supporting Information for

Farmland practices are driving bird populations decline across Europe

Stanislas Rigal, Vasilis Dakos, Hany Alonso, Ainārs Auniņš, Zoltán Benkő, Lluis Brotons, Tomasz Chodkiewicz, Przemysław Chylarecki, Elisabetta de Carli, Juan Carlos del Moral, Cristian Domșa, Virginia Escandell, Benoît Fontaine, Ruud Foppen, Richard Gregory, Sarah Harris, Sergi Herrando, Magne Husby, Christina Ieronymidou, Frederic Jiguet, John Kennedy, Alena Klvaňová, Primož Kmecl, Lechosław Kuczyński, Petras Kurlavičius, John Atle Kålås, Aleksi Lehikoinen, Åke Lindström, Romain Lorrillière, Charlotte Moshøj, Renno Nellis, David Noble, Daniel Palm Eskildsen, Jean-Yves Paquet, Mathieu Pelissié, Clara Pladevall, Danae Portolou, Jiří Reif, Hans Schmid, Benjamin Seaman, Zoltán D. Szabo, Tibor Szép, Guido Tellini Florenzano, Norbert Teufelbauer, Sven Trautmann, Chris van Turnhout, Zdeněk Vermouzek, Thomas Vikstrøm, Petr Voříšek, Anne Weiserbs, Vincent Devictor.

Corresponding author: Stanislas Rigal

Email: stanislas.rigal@ens-lyon.fr

This PDF file includes:

Supplementary Information Appendix 1

Figure S1

Supporting text

Table S1

Supplementary Information Appendix 2

Table S2 to S3

Figure S2

Supplementary Information Appendix 3

Figures S3 to S4

Supplementary Information Appendix 4

Figure S5

Supplementary Information Appendix 5

Tables S4 to S10

Supplementary Information Appendix 6

Figure S6

Supplementary Information Appendix 7

Tables S11 to S15

Figure S7

Supplementary Information Appendix 8
Figure S8
SI References

Supplementary Information Appendix 1

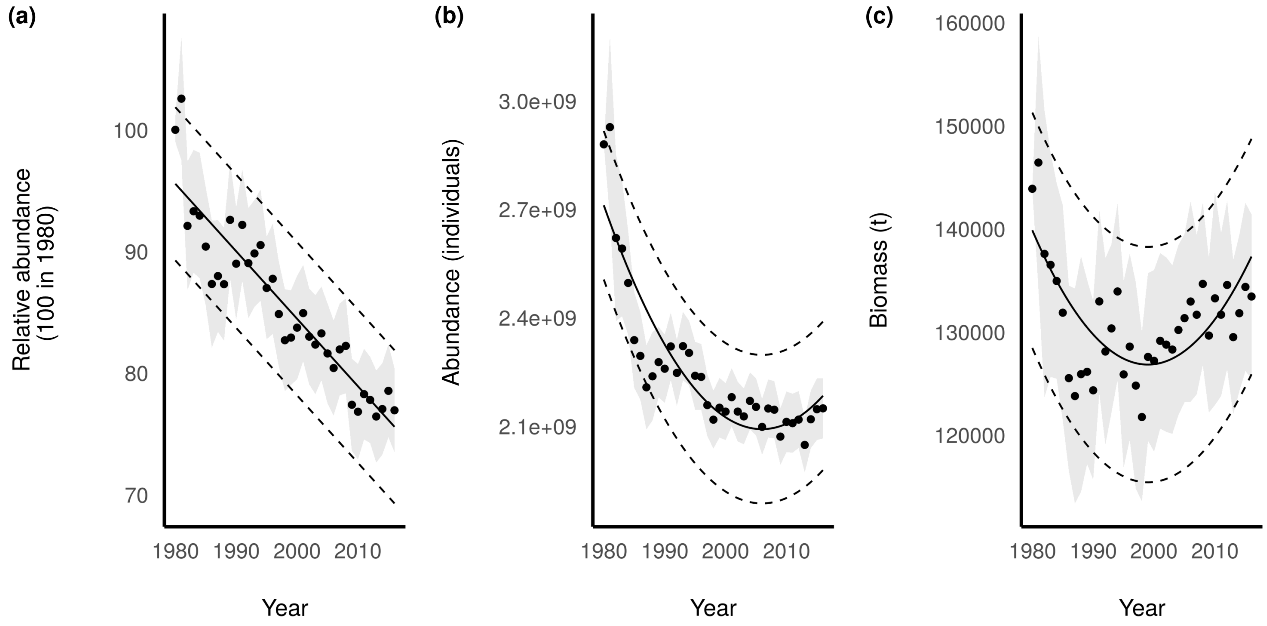

Figure S1: Multispecies index, abundance and biomass (see methods below) of all the 115 common bird species (used for supranational indices among the 170 species) between 1980 and 2016. a) The relative abundance multispecies index is calculated by attributing a common weight to each species whatever their abundance or biomass and shows that a majority of species have been declining. b) Abundance corresponds to the number of individuals and it is discussed in the paper and used as referenced in this figure. Biomass was obtained by multiplying the average weight of each species by the abundance. c) Biomass trajectory shows a convex shape interpreted as an increase of protected and rare birds (often heavy species) whereas more common (and light) ones were decreasing.

Supporting text: Supplementary Method for species abundance

In addition to the Species Abundance Index (SAI) which corresponds to a relative value, estimated numbers of breeding pairs by countries were manually extracted from the European Red List of Birds fact-sheets available on Birdlife Data Zone to evaluate annual absolute abundance (1). The geometric mean of the minimum and maximum number of species pairs was multiplied by two to obtain the number of individuals. This gave us an estimate of each countrywide species population size in a breeding season. Population estimates stem from survey data with average starting and ending years being 2007 and 2011 respectively. They are considered as proxies of the population size at the ending year

(*e.g.* population of *Anthus trivialis* in France was estimated at 529 150 individuals at the ending year 2012 in Birdlife). To obtain population size for each year, we used the SAI corresponding to relative variations in abundance compared to a reference year (*e.g.* 1989 for *A. trivialis* in France). SAI values are scaled with the reference year value set at 100. The population size at the ending year was divided by the SAI value at the ending year (*e.g.* in 2012 for *A. trivialis* in France, SAI=72 (se=17)) (or at the first or last year, if population size was estimated before or after the period covered by the PECBMS, respectively). This resulted in a weighing factor (*e.g.* here 529 150/72=7349) which was then used to multiply SAI values to get the absolute abundance (and its standard errors) of the population for each year. This was repeated for each of the 170 species and in each of the 28 countries.

Period	Slope	Pvalue	Significant decline
1980-2016	$-1.5.10^{7}$	$1.1.10^{-09}$	Yes
1981-2016	$-1.2.10^{7}$	$1.4.10^{-08}$	Yes
1982-2016	$-1.0.10^{7}$	$2.4.10^{-08}$	Yes
1983-2016	$-8.9.10^{6}$	$1.8.10^{-07}$	Yes
1984-2016	$-7.8.10^{6}$	$8.6.10^{-07}$	Yes
1985-2016	$-6.7.10^{6}$	$1.5.10^{-05}$	Yes
1986-2016	$-6.5.10^{6}$	$5.7.10^{-05}$	Yes
1987-2016	$-6.4.10^{6}$	$1.9.10^{-04}$	Yes
1988-2016	$-7.0.10^{6}$	$2.3.10^{-05}$	Yes
1989-2016	$-7.2.10^{6}$	$2.2.10^{-06}$	Yes
1990-2016	$-7.0.10^{6}$	$8.4.10^{-06}$	Yes
1991-2016	$-7.1.10^{6}$	$7.4.10^{-06}$	Yes
1992-2016	$-6.5.10^{6}$	$6.0.10^{-05}$	Yes
1993-2016	$-6.4.10^{6}$	$2.0.10^{-04}$	Yes
1994-2016	$-5.2.10^{6}$	$1.3.10^{-03}$	Yes
1995-2016	$-4.2.10^{6}$	$8.4.10^{-03}$	Yes
1996-2016	$-3.3.10^{6}$	$4.3.10^{-02}$	Yes
1997-2016	$-3.3.10^{6}$	$4.3.10^{-02}$	Yes
1998-2016	$-2.4.10^{6}$	$1.0.10^{-01}$	No
1999-2016	$-2.4.10^{6}$	$1.3.10^{-01}$	No
2000-2016	$-2.5.10^{6}$	$1.4.10^{-01}$	No
2001-2016	$-2.8.10^{6}$	$1.5.10^{-01}$	No
2002-2016	$-2.8.10^{6}$	$1.7.10^{-01}$	No
2003-2016	$-2.1.10^{6}$	$3.7.10^{-01}$	No
2004-2016	$-1.7.10^{6}$	$4.9.10^{-01}$	No
2005-2016	$-2.6.10^{6}$	$3.8.10^{-01}$	No
2006-2016	$-5.5.10^{4}$	$7.1.10^{-01}$	No

Table S1: Slope of a linear regression of the overall bird abundance trajectory and time period considered.

Supplementary Information Appendix 2

1. Detailed results of PLS

	Component 1	Component 2
R^2	0.01	0.01
High-input farm cover	-0.57	0.01
High-input farm cover trend	-0.32	-0.54
Forest cover	0.29	-0.42
Forest cover trend	0.03	-0.08
Urbanisation	-0.38	0.36
Urbanisation trend	-0.55	-0.01
Temperature	0.14	0.52
Temperature trend	-0.08	-0.34

Table S2: Detailed results of the Partial Least Square Regression between bird population trends and pressures. The variance explained by each of the two selected component is given by R^2 and the normed weight of each explanatory variable in each component is provided.

2. Interaction terms in PLS

Interaction term	Mean of interaction effect	CI inf	CI sup
High input farm * trend in high input farm	0.045	0.019	0.073
Forest cover * trend in forest cover	0.009	0	0.018
Urbanisation cover * trend in urbanisation cover	-0.004	-0.018	0.009
Temperature * trend in temperature	0.013	0.005	0.019

Table S3: Interaction effects between pressures and pressure trends. Interaction effects are computed one by one (as the addition of an interaction terms change the interpretation of effects of other terms) via a Partial Least Square Regression between national bird species trend and the four explanatory variables (high-input farm cover and its temporal trend, forest cover and its temporal trend, urbanisation and its temporal trend, and temperature and its temporal trend). Lower and upper boundaries of bias-corrected and accelerated confidence interval (CI) are provided.

Overall, adding interactive terms does not change the sign, only the magnitude of the main terms. The interactive terms are only significant for high input cover and temperature (Tab S3). They show that the negative effect of high input cover change is less important when high input cover is already high, and the negative effect of temperature trend is less important when the average temperature is already high.

3. Effect of another component of agricultural intensification: farm size

We obtained data on farm size from the Farm structure dataset available from the European Statistics (2). We calculated the ratio of the number of small farms (< 5 ha) to the number of large farms (> 50 ha) in order to obtain a value that is independent of the total number of farms and the utilised agricultural area of each country. By incorporating this term into the PLS analysis (Fig. S2), we found a positive effect of having a high number of small farms compared to the number of large farms.

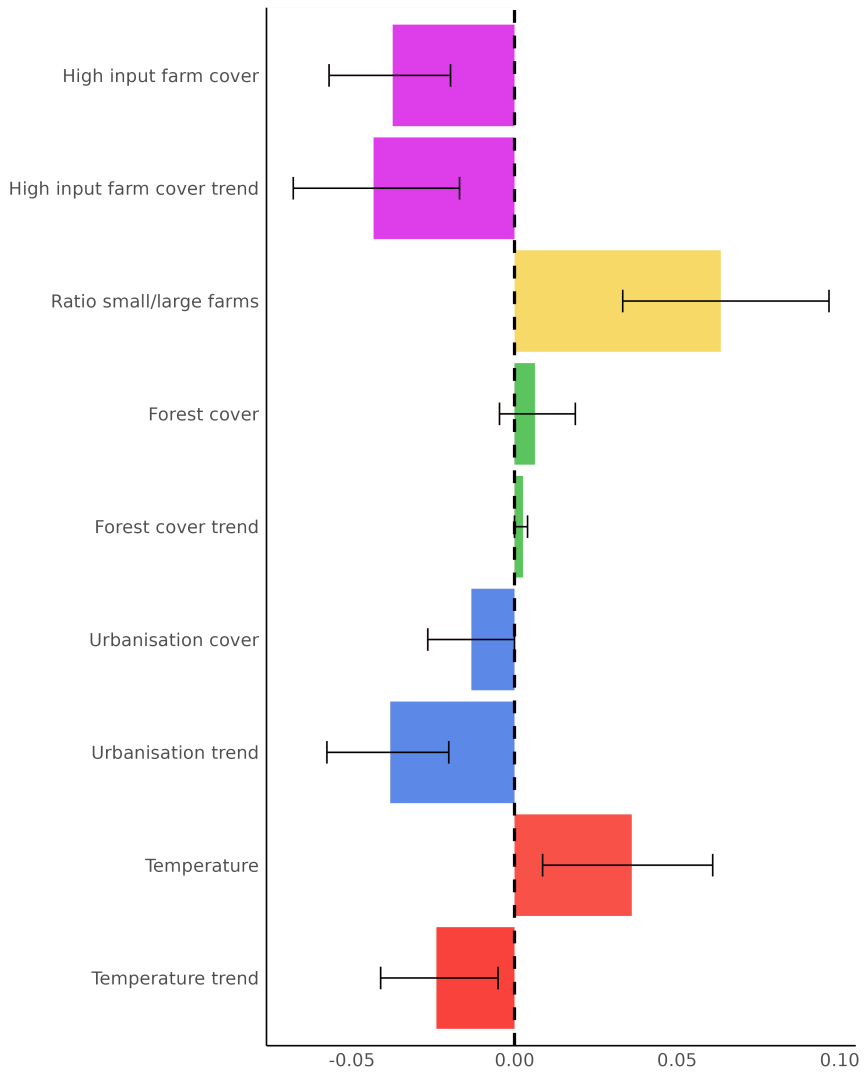

Figure S2: Relationship between anthropogenic pressure and bird trends and time-series. a) Relative effects of high-input farm cover, farm size, forest cover, urbanisation, temperature and their trends on bird trends (1996-2016, 141 species) obtained by partial least square regression (PLS). Bias-corrected and accelerated confidence intervals are displayed. The ratio of small/large farms corresponds to the number of farms < 5 ha to the number of farms > 50 ha.

Supplementary Information Appendix 3

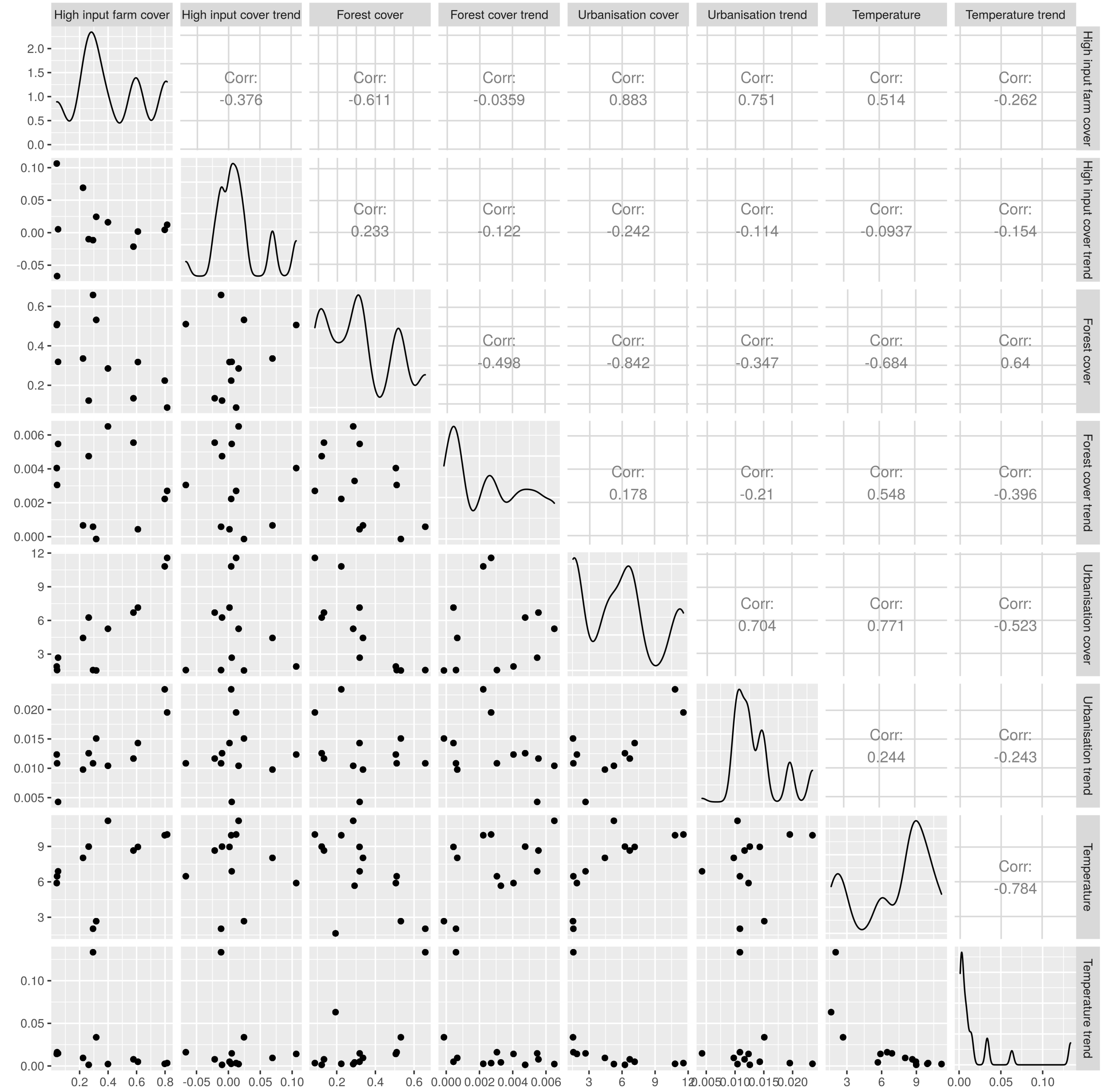

Figure S3: Density plots and correlations between anthropogenic pressures (means and trends): high-input farm cover, forest cover, urbanisation and temperature change. Each dot represents a country.

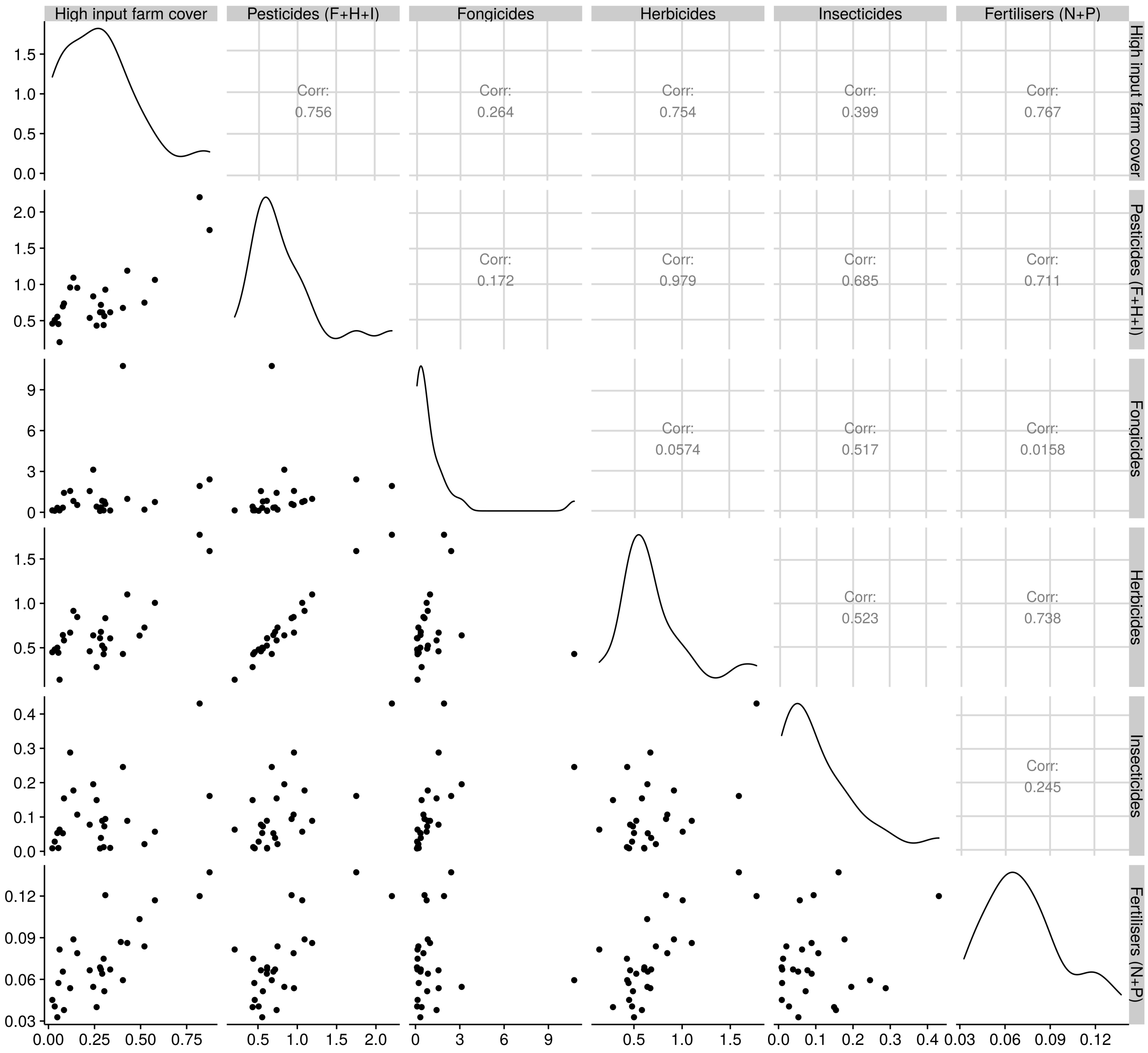

Figure S4: Correlations between high-input farm cover, pesticide sales and fertiliser consumption for European countries. Pesticides correspond to the total amount of herbicides, insecticides, fungicides sold per hectare. Fertilisers correspond to the total amount of nitrogen and phosphorus applied per hectare. Each dot represents a country.

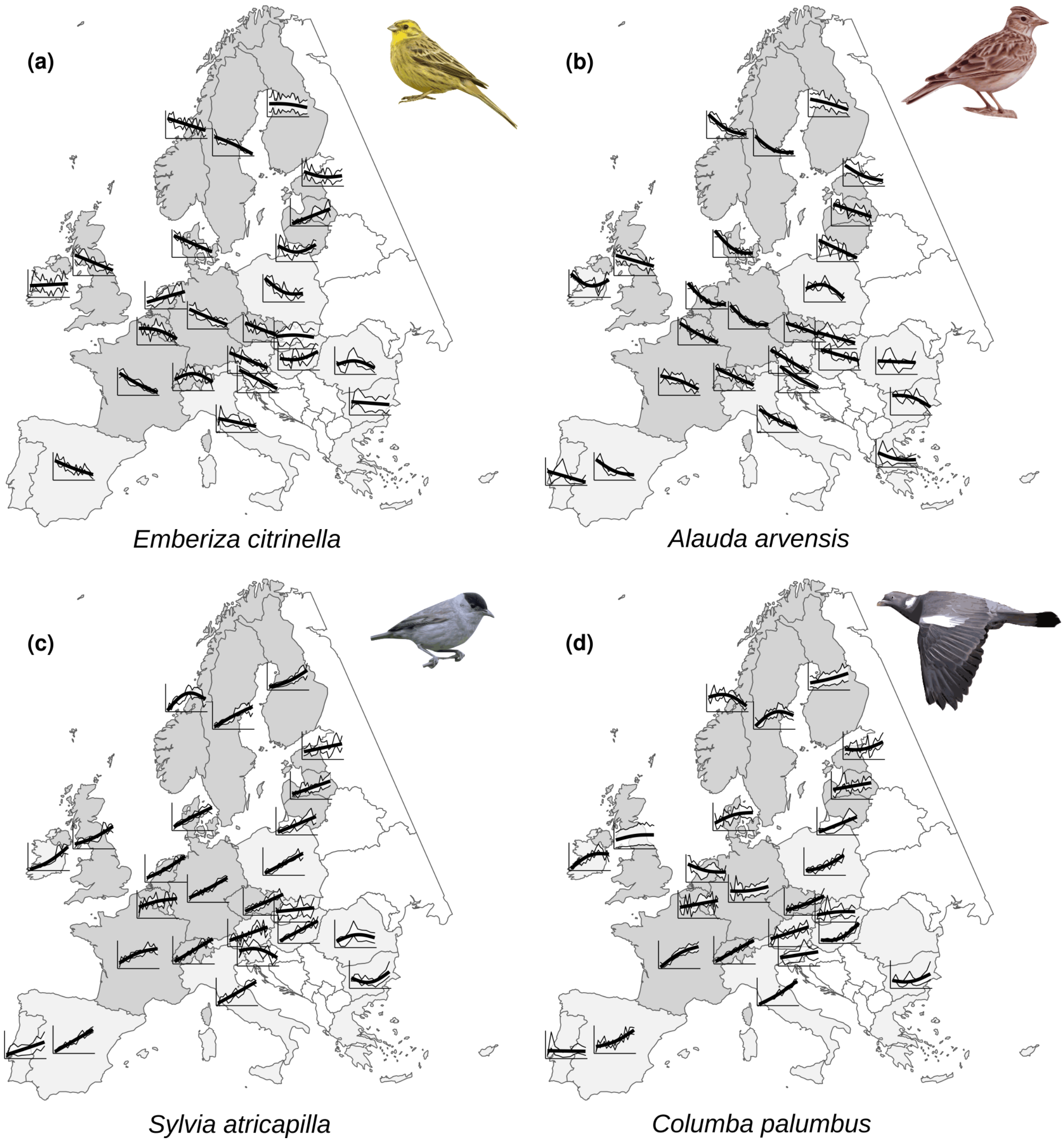

Figure S5: National trajectories of a) the yellowhammer *Emberiza citrinella, b)* the Eurasian skylark *Alauda arvensis*, c) the Eurasian blackcap *Sylvia atricapilla* and d) the common wood pigeon *Columba palumbus* among the European countries over the last decades. The main trajectory is shown by a black line and data with standard errors are shown in white. *E. citrinella* had mostly negative trends but in some countries (*e.g.* the Netherlands or Latvia) populations increased. On the contrary, *A. arvensis* only experienced negative trend among all countries. *S. atricapilla* and *C. palumbus* are

increasing in nearly all countries. Countries in grey are involved in the PanEuropean Common Bird Monitoring Scheme (PECBMS). Data are available from at least from 1996 to 2016 in 14 countries (medium grey) and after 1996 for others (light grey). Among the 124 species present in at least 2 countries, 17 species had a similar trend across countries (always negative) and 107 had different trends depending on the country.

Supplementary Information Appendix 5

Species names	Selected for multispecies indices	Selected for species trends	PECBMS habitat	Habitat generalist	Urban dweller	Cold vs. hot dwellers
Acanthis flammea	Yes	Yes	Other			Cold
Accipiter nisus	Yes	Yes	Forest	Yes	Yes	
Acrocephalus arundinaceus		Yes	Other			
Acrocephalus palustris	Yes	Yes	Other			
Acrocephalus schoenobaenus	Yes	Yes	Other			
Acrocephalus scirpaceus	Yes	Yes	Other			Hot
Actitis hypoleucos	Yes	Yes	Other			Cold
Aegithalos caudatus	Yes	Yes	Other			Hot
Alauda arvensis	Yes	Yes	Farmland			
Alcedo atthis	Yes	Yes	Other			Hot
Alectoris rufa			Farmland			
Anas platyrhynchos	Yes	Yes	Other			
Anthus campestris		Yes	Farmland			
Anthus pratensis	Yes	Yes	Farmland			Cold
Anthus trivialis	Yes	Yes	Forest	Yes		Cold
Apus apus	Yes	Yes	Other		Yes	
Ardea cinerea	Yes	Yes	Other	Yes		Hot
Bombycilla garrulus		Yes	Forest			
Bonasa bonasia	Yes	Yes	Forest			Cold
Bubulcus ibis			Farmland			
Burhinus oedicnemus			Farmland			
Buteo buteo	Yes	Yes	Other			Hot
Calandrella brachydactyla			Farmland			
Calcarius lapponicus	Yes	Yes	Other			Cold
Carduelis carduelis	Yes	Yes	Other			Hot
Carduelis citrinella			Forest			
Carpodacus erythrinus	Yes	Yes	Other	Yes		Cold
Cecropis daurica			Other			
Certhia brachydactyla		Yes	Forest			
Certhia familiaris	Yes	Yes	Forest			Cold
Cettia cetti		Yes	Other			
Chloris chloris	Yes	Yes	Other			Hot
Ciconia ciconia	Yes	Yes	Farmland		Yes	Hot
Circus aeruginosus	Yes	Yes	Other	Yes		Hot
Cisticola juncidis			Other			

Clamator glandarius			Other			
Coccothraustes coccothraustes	Yes	Yes	Forest			Hot
Columba oenas	Yes	Yes	Forest	Yes		
Columba palumbus	Yes	Yes	Other	Yes	Yes	
Corvus corax	Yes	Yes	Other			
Corvus corone	Yes	Yes	Other		Yes	
Corvus frugilegus	Yes	Yes	Farmland		Yes	Hot
Corvus monedula	Yes	Yes	Other		Yes	Hot
Cuculus canorus	Yes	Yes	Other	Yes		
Cyanistes caeruleus	Yes	Yes	Other			Hot
Cyanopica cyanus			Forest			
Cygnus olor	Yes	Yes	Other			
Delichon urbicum	Yes	Yes	Other		Yes	
Dendrocopos major	Yes	Yes	Other		Yes	
Dendrocopos syriacus			Other		Yes	
Dendrocoptes medius		Yes	Forest			
Dryobates minor	Yes	Yes	Forest			
Dryocopus martius	Yes	Yes	Forest			Cold
Egretta garzetta			Other			
Emberiza calandra	Yes	Yes	Farmland			Hot
Emberiza cia			Other			
Emberiza cirlus		Yes	Farmland			
Emberiza citrinella	Yes	Yes	Farmland			
Emberiza hortulana	Yes	Yes	Farmland	Yes		Hot
Emberiza melanocephala			Farmland			
Emberiza rustica	Yes	Yes	Forest			Cold
Emberiza schoeniclus	Yes	Yes	Other			Cold
Erithacus rubecula	Yes	Yes	Other	Yes	Yes	
Falco tinnunculus	Yes	Yes	Farmland	Yes		
Ficedula albicollis		Yes	Forest		Yes	
Ficedula hypoleuca	Yes	Yes	Forest		Yes	Cold
Fringilla coelebs	Yes	Yes	Other			
Fringilla montifringilla	Yes	Yes	Other			Cold
Fulica atra	Yes	Yes	Other			Hot
Galerida cristata		Yes	Farmland		Yes	
Galerida theklae			Farmland			
Gallinago gallinago	Yes	Yes	Other			Cold
Gallinula chloropus	Yes	Yes	Other			Hot
Garrulus glandarius	Yes	Yes	Forest			Hot

Grus grus	Yes	Yes	Other			Cold
Haematopus ostralegus	Yes	Yes	Other	Yes		Cold
Hippolais icterina	Yes	Yes	Other			
Hippolais polyglotta		Yes	Other			
Hirundo rustica	Yes	Yes	Farmland		Yes	
Iduna pallida			Other			
Jynx torquilla	Yes	Yes	Other			
Lanius collurio	Yes	Yes	Farmland			Hot
Lanius minor			Farmland			
Lanius senator			Farmland			
Larus ridibundus	Yes	Yes	Other	Yes		Cold
Limosa limosa		Yes	Farmland			
Linaria cannabina	Yes	Yes	Farmland			Hot
Locustella fluviatilis		Yes	Other			
Locustella naevia	Yes	Yes	Other			
Lophophanes cristatus	Yes	Yes	Forest			
Lullula arborea	Yes	Yes	Other	Yes		Hot
Luscinia luscinia	Yes	Yes	Other			
Luscinia megarhynchos	Yes	Yes	Other	Yes		Hot
Luscinia svecica	Yes	Yes	Other			Cold
Lyrurus tetrix	Yes	Yes	Other			Cold
Melanocorypha calandra			Farmland			
Merops apiaster		Yes	Other			
Motacilla alba	Yes	Yes	Other	Yes	Yes	
Motacilla cinerea	Yes	Yes	Other			Hot
Motacilla flava	Yes	Yes	Farmland			
Muscicapa striata	Yes	Yes	Other	Yes	Yes	
Nucifraga caryocatactes	Yes	Yes	Forest			Cold
Numenius arquata	Yes	Yes	Other			Cold
Numenius phaeopus		Yes	Other			
Oenanthe cypriaca			NA			
Oenanthe hispanica			Farmland			
Oenanthe oenanthe	Yes	Yes	Other	Yes		
Oriolus oriolus		Yes	Other			
Parus major	Yes	Yes	Other	Yes	Yes	
Passer domesticus	Yes	Yes	Other	Yes	Yes	
Passer montanus	Yes	Yes	Farmland		Yes	Hot
Perdix perdix	Yes	Yes	Farmland			Hot
Periparus ater	Yes	Yes	Forest			
Petronia petronia			Farmland			

Phasianus colchicus	Yes	Yes	Other			Hot
Phoenicurus ochruros		Yes	Other		Yes	
Phoenicurus phoenicurus	Yes	Yes	Forest	Yes		
Phylloscopus bonelli		Yes	Forest			
Phylloscopus collybita	Yes	Yes	Forest			
Phylloscopus sibilatrix	Yes	Yes	Forest			
Phylloscopus trochilus	Yes	Yes	Other			Cold
Pica pica	Yes	Yes	Other	Yes	Yes	
Picus canus		Yes	Forest			
Picus viridis	Yes	Yes	Other	Yes		Hot
Pluvialis apricaria	Yes	Yes	Other			Cold
Podiceps cristatus	Yes	Yes	Other			Hot
Poecile montanus	Yes	Yes	Forest			Cold
Poecile palustris	Yes	Yes	Forest			
Prunella modularis	Yes	Yes	Other	Yes	Yes	Cold
Ptyonoprogne rupestris			Other			
Pyrrhocorax pyrrhocorax			Other			
Pyrrhula pyrrhula	Yes	Yes	Forest			Cold
Regulus ignicapilla		Yes	Forest			
Regulus regulus	Yes	Yes	Forest			Cold
Saxicola rubetra	Yes	Yes	Farmland			Cold
Saxicola torquatus		Yes	Farmland			
Serinus serinus		Yes	Farmland		Yes	
Sitta europaea	Yes	Yes	Forest			Hot
Spinus spinus	Yes	Yes	Forest			Cold
Streptopelia decaocto	Yes	Yes	Other		Yes	Hot
Streptopelia turtur	Yes	Yes	Farmland	Yes		Hot
Sturnus unicolor			Farmland		Yes	
Sturnus vulgaris	Yes	Yes	Farmland		Yes	
Sylvia atricapilla	Yes	Yes	Other	Yes		Hot
Sylvia borin	Yes	Yes	Other			
Sylvia cantillans		Yes	Other			
Sylvia communis	Yes	Yes	Farmland			Hot
Sylvia curruca	Yes	Yes	Other	Yes		
Sylvia hortensis		Yes	Other			
Sylvia melanocephala		Yes	Other			
Sylvia melanothorax			NA			
Sylvia nisoria		Yes	Other			
Sylvia undata			Other			
Tachybaptus ruficollis	Yes	Yes	Other			Hot

Tadorna tadorna	Yes	Yes	Other			
Tetrax tetrax			Farmland			
Tringa erythropus			Other			
Tringa glareola	Yes	Yes	Other			Cold
Tringa nebularia	Yes	Yes	Other			Cold
Tringa ochropus	Yes	Yes	Forest			Cold
Tringa totanus	Yes	Yes	Other			Cold
Troglodytes troglodytes	Yes	Yes	Other	Yes		
Turdus iliacus	Yes	Yes	Other			Cold
Turdus merula	Yes	Yes	Other	Yes	Yes	
Turdus philomelos	Yes	Yes	Other	Yes		Hot
Turdus pilaris	Yes	Yes	Other			Cold
Turdus torquatus			Other			
Turdus viscivorus	Yes	Yes	Forest			
Upupa epops		Yes	Farmland			
Vanellus vanellus	Yes	Yes	Farmland			

Table S4: List of the 170 European common bird species. Species habitat classification is shown according to the PanEuropean Common Bird Monitoring Scheme (PECBMS) classification. Generalist species are those with the lowest habitat specialisation index (SSI). Urban dwellers are species with a positive synanthropic index. Hot dwellers are the species with the highest STI (30%) and cold dwellers with the lowest STI (30%). Bolded species are the 115 species used for supranational multispecies indices, i.e. species monitored for the first time before 1981.

Country	Number of species
Austria	90
Belgium	81
Bulgaria	56
Cyprus	27
Czech Republic	108
Denmark	88
Estonia	80
Finland	86
France	129
Germany	111
Greece	45
Hungary	90
Ireland	54
Italy	114
Latvia	84
Lithuania	61
Luxembourg	40
Netherlands	102
Norway	72
Poland	111
Portugal	84
Romania	31
Slovakia	90
Slovenia	62
Spain	113
Sweden	113
Switzerland	103
United Kingdom	89

Table S5: Number of species among the 170 that are monitored in each country.

Species names	Austria	Belgium	Bulgaria	Cyprus	Czech Republic	Denmark	Estonia	Finland	France	Germany	Greece	Hungary	Italy	Ireland	Latvia	Lithuania	Luxembourg	Netherlands	Norway	Poland	Portugal	Romania	Slovakia	Slovenia	Spain	Sweden	Switzerland	UK
Alauda arvensis	Yes	Yes	Yes		Yes	Yes	Yes	Yes	Yes	Yes	Yes	Yes	Yes	Yes	Yes	Yes	Yes	Yes	Yes	Yes	Yes	Yes	Yes	Yes	Yes	Yes	Yes	Yes
Anthus pratensis		Yes			Yes	Yes	Yes	Yes	Yes	Yes				Yes	Yes	Yes		Yes	Yes	Yes						Yes	Yes	Yes
Ciconia ciconia			Yes		Yes				Yes	Yes	Yes	Yes			Yes	Yes		Yes		Yes	Yes		Yes		Yes		Yes	
Corvus frugilegus		Yes			Yes	Yes			Yes	Yes		Yes		Yes				Yes		Yes			Yes			Yes	Yes	Yes
Emberiza calandra	Yes	Yes	Yes	Yes	Yes	Yes			Yes	Yes	Yes	Yes	Yes					Yes		Yes	Yes	Yes	Yes	Yes	Yes			Yes
Emberiza citrinella	Yes	Yes	Yes		Yes	Yes	Yes	Yes	Yes	Yes		Yes	Yes	Yes	Yes	Yes	Yes	Yes	Yes	Yes		Yes	Yes	Yes	Yes	Yes	Yes	Yes
Emberiza hortulana			Yes					Yes	Yes	Yes										Yes					Yes	Yes		
Falco tinnunculus	Yes	Yes	Yes	Yes	Yes	Yes			Yes	Yes	Yes	Yes	Yes	Yes				Yes		Yes	Yes		Yes	Yes	Yes	Yes	Yes	Yes
Hirundo rustica	Yes	Yes	Yes	Yes	Yes	Yes	Yes	Yes	Yes	Yes	Yes	Yes	Yes	Yes	Yes	Yes	Yes	Yes	Yes	Yes	Yes		Yes	Yes	Yes	Yes	Yes	Yes
Lanius collurio	Yes	Yes	Yes		Yes	Yes	Yes	Yes	Yes	Yes	Yes	Yes	Yes		Yes			Yes		Yes		Yes	Yes	Yes	Yes	Yes	Yes	
Linaria cannabina	Yes	Yes		Yes	Yes	Yes	Yes		Yes	Yes	Yes	Yes	Yes	Yes	Yes	Yes	Yes	Yes		Yes	Yes		Yes	Yes	Yes	Yes	Yes	Yes
Motacilla flava		Yes	Yes		Yes	Yes		Yes	Yes	Yes		Yes	Yes		Yes	Yes		Yes	Yes	Yes	Yes	Yes	Yes	Yes	Yes	Yes	Yes	Yes
Passer montanus	Yes	Yes	Yes		Yes	Yes	Yes		Yes	Yes	Yes	Yes	Yes		Yes	Yes		Yes		Yes	Yes	Yes	Yes	Yes	Yes	Yes	Yes	Yes
Perdix perdix	Yes	Yes			Yes	Yes			Yes	Yes		Yes						Yes		Yes								Yes
Saxicola rubetra	Yes				Yes	Yes	Yes	Yes	Yes	Yes		Yes	Yes		Yes	Yes		Yes	Yes	Yes		Yes	Yes	Yes	Yes	Yes	Yes	Yes
Streptopelia turtur	Yes	Yes	Yes	Yes	Yes		Yes		Yes	Yes	Yes	Yes	Yes		Yes	Yes		Yes		Yes	Yes	Yes	Yes	Yes	Yes		Yes	Yes
Sturnus vulgaris	Yes	Yes	Yes		Yes	Yes	Yes	Yes	Yes	Yes	Yes	Yes	Yes	Yes	Yes	Yes	Yes	Yes	Yes	Yes		Yes	Yes	Yes	Yes	Yes	Yes	Yes
Sylvia communis	Yes	Yes	Yes		Yes	Yes	Yes	Yes	Yes	Yes	Yes	Yes	Yes	Yes	Yes	Yes		Yes	Yes	Yes		Yes	Yes	Yes	Yes	Yes	Yes	Yes
Vanellus vanellus	Yes	Yes			Yes	Yes	Yes	Yes	Yes	Yes		Yes	Yes		Yes	Yes		Yes	Yes	Yes			Yes	Yes		Yes		Yes

Table S6: 19 species selected for the computation of the farmland multispecies index for each county.

Species names	Austria	Belgium	Bulgaria	Cyprus	Czech Republic	Denmark	Estonia	Finland	France	Germany	Greece	Hungary	Italy	Ireland	Latvia	Lithuania	Luxembourg	Netherlands	Norway	Poland	Portugal	Romania	Slovakia	Slovenia	Spain	Sweden	Switzerland	UK
Accipiter nisus		Yes			Yes	Yes			Yes	Yes		Yes	Yes	Yes	Yes			Yes		Yes					Yes	Yes	Yes	Yes
Anthus trivialis	Yes	Yes			Yes	Yes	Yes	Yes	Yes	Yes		Yes	Yes		Yes	Yes	Yes	Yes	Yes	Yes		Yes	Yes	Yes	Yes	Yes	Yes	Yes
Bonasa bonasia							Yes	Yes							Yes											Yes	Yes	Yes
Certhia familiaris	Yes	Yes			Yes	Yes	Yes	Yes	Yes	Yes			Yes	Yes	Yes					Yes			Yes			Yes	Yes	
Coccothraustes coccothraustes	Yes	Yes	Yes		Yes	Yes	Yes		Yes	Yes		Yes	Yes		Yes	Yes	Yes	Yes		Yes	Yes		Yes	Yes	Yes	Yes	Yes	
Columba oenas	Yes	Yes			Yes	Yes	Yes	Yes	Yes	Yes		Yes		Yes	Yes			Yes		Yes			Yes		Yes	Yes	Yes	Yes
Dryobates minor	Yes	Yes			Yes		Yes		Yes	Yes		Yes			Yes			Yes		Yes	Yes		Yes			Yes	Yes	Yes
Dryocopus martius	Yes	Yes			Yes	Yes	Yes	Yes	Yes	Yes		Yes	Yes		Yes	Yes		Yes	Yes	Yes			Yes	Yes	Yes	Yes	Yes	
Emberiza rustica								Yes																		Yes		
Ficedula hypoleuca					Yes	Yes	Yes	Yes	Yes	Yes					Yes	Yes		Yes	Yes	Yes					Yes	Yes	Yes	Yes
Garrulus glandarius	Yes	Yes	Yes		Yes	Yes	Yes	Yes	Yes	Yes	Yes	Yes	Yes		Yes	Yes	Yes	Yes	Yes	Yes	Yes	Yes	Yes	Yes	Yes	Yes	Yes	Yes
Lophophanes cristatus	Yes	Yes			Yes	Yes	Yes	Yes	Yes	Yes			Yes		Yes			Yes	Yes	Yes	Yes		Yes		Yes	Yes	Yes	
Nucifraga caryocatactes					Yes		Yes		Yes				Yes		Yes											Yes	Yes	
Periparus ater	Yes	Yes		Yes	Yes	Yes		Yes	Yes	Yes		Yes	Yes	Yes	Yes		Yes	Yes	Yes	Yes	Yes		Yes		Yes	Yes	Yes	Yes
Phoenicurus phoenicurus	Yes	Yes			Yes	Yes	Yes	Yes	Yes	Yes			Yes		Yes			Yes	Yes	Yes	Yes		Yes	Yes	Yes	Yes	Yes	Yes
Phylloscopus collybita	Yes	Yes	Yes		Yes	Yes	Yes	Yes	Yes	Yes		Yes	Yes	Yes	Yes	Yes	Yes	Yes	Yes	Yes		Yes	Yes	Yes		Yes	Yes	Yes
Phylloscopus sibilatrix	Yes	Yes			Yes	Yes	Yes	Yes	Yes	Yes		Yes	Yes		Yes	Yes		Yes		Yes			Yes			Yes	Yes	Yes
Poecile montanus	Yes	Yes			Yes		Yes	Yes	Yes	Yes			Yes		Yes			Yes	Yes	Yes			Yes			Yes	Yes	Yes
Poecile palustris	Yes	Yes			Yes	Yes	Yes		Yes	Yes		Yes	Yes		Yes		Yes	Yes		Yes			Yes	Yes		Yes	Yes	Yes
Pyrrhula pyrrhula	Yes	Yes			Yes	Yes	Yes	Yes	Yes	Yes			Yes	Yes	Yes			Yes	Yes	Yes			Yes		Yes	Yes	Yes	Yes
Regulus regulus	Yes	Yes			Yes	Yes	Yes	Yes	Yes	Yes			Yes	Yes	Yes		Yes	Yes	Yes	Yes			Yes		Yes	Yes	Yes	Yes
Sitta europaea	Yes	Yes			Yes	Yes	Yes		Yes	Yes		Yes	Yes		Yes	Yes	Yes	Yes	Yes	Yes	Yes		Yes	Yes	Yes	Yes	Yes	Yes
Spinus spinus	Yes				Yes	Yes	Yes	Yes		Yes			Yes		Yes			Yes	Yes	Yes			Yes			Yes	Yes	Yes
Tringa ochropus							Yes	Yes							Yes				Yes							Yes		
Turdus viscivorus	Yes	Yes			Yes	Yes	Yes	Yes	Yes	Yes		Yes	Yes	Yes	Yes		Yes	Yes	Yes	Yes	Yes		Yes	Yes	Yes	Yes	Yes	Yes

Table S7: 25 species selected for the computation of the forest multispecies index for each county.

Species names	Austria	Belgium	Bulgaria	Cyprus	Czech Republic	Denmark	Estonia	Finland	France	Germany	Greece	Hungary	Italy	Ireland	Latvia	Lithuania	Luxembourg	Netherlands	Norway	Poland	Portugal	Romania	Slovakia	Slovenia	Spain	Sweden	Switzerland	UK
Accipiter nisus		Yes			Yes	Yes			Yes	Yes		Yes	Yes	Yes	Yes			Yes		Yes					Yes	Yes	Yes	Yes
Apus apus	Yes	Yes	Yes	Yes	Yes	Yes	Yes	Yes	Yes	Yes	Yes		Yes	Yes					Yes	Yes	Yes		Yes		Yes	Yes	Yes	Yes
Ciconia ciconia			Yes		Yes				Yes	Yes	Yes	Yes			Yes	Yes		Yes		Yes	Yes		Yes		Yes		Yes	
Columba palumbus	Yes	Yes	Yes	Yes	Yes	Yes	Yes	Yes	Yes	Yes		Yes	Yes	Yes	Yes	Yes	Yes	Yes	Yes	Yes	Yes		Yes	Yes	Yes	Yes	Yes	Yes
Corvus corone	Yes	Yes	Yes	Yes	Yes	Yes	Yes	Yes	Yes	Yes	Yes	Yes	Yes	Yes	Yes	Yes	Yes	Yes	Yes	Yes	Yes		Yes	Yes	Yes	Yes	Yes	Yes
Corvus frugilegus		Yes			Yes	Yes			Yes	Yes		Yes		Yes				Yes		Yes			Yes			Yes	Yes	Yes
Coloeus monedula	Yes	Yes	Yes	Yes	Yes	Yes	Yes	Yes	Yes	Yes	Yes		Yes	Yes	Yes	Yes	Yes	Yes		Yes			Yes	Yes	Yes	Yes	Yes	Yes
Delichon urbicum	Yes	Yes	Yes	Yes	Yes	Yes	Yes	Yes	Yes	Yes	Yes	Yes	Yes	Yes		Yes		Yes	Yes	Yes	Yes		Yes	Yes	Yes	Yes	Yes	Yes
Dendrocopos major	Yes	Yes	Yes		Yes	Yes	Yes	Yes	Yes	Yes		Yes	Yes		Yes	Yes	Yes	Yes	Yes	Yes	Yes	Yes	Yes	Yes	Yes	Yes	Yes	Yes
Erithacus rubecula	Yes	Yes	Yes		Yes	Yes	Yes	Yes	Yes	Yes		Yes	Yes	Yes	Yes	Yes	Yes	Yes	Yes	Yes	Yes	Yes	Yes	Yes	Yes	Yes	Yes	Yes
Ficedula hypoleuca					Yes	Yes	Yes	Yes	Yes	Yes					Yes	Yes		Yes	Yes	Yes					Yes	Yes	Yes	Yes
Hirundo rustica	Yes	Yes	Yes	Yes	Yes	Yes	Yes	Yes	Yes	Yes	Yes	Yes	Yes	Yes	Yes	Yes	Yes	Yes	Yes	Yes	Yes		Yes	Yes	Yes	Yes	Yes	Yes
Motacilla alba	Yes	Yes			Yes	Yes	Yes	Yes	Yes	Yes		Yes	Yes	Yes	Yes	Yes		Yes	Yes	Yes	Yes	Yes	Yes	Yes	Yes	Yes	Yes	Yes
Muscicapa striata	Yes	Yes			Yes	Yes	Yes	Yes	Yes	Yes		Yes	Yes	Yes	Yes	Yes		Yes	Yes	Yes			Yes	Yes	Yes	Yes	Yes	Yes
Parus major	Yes	Yes	Yes	Yes	Yes	Yes	Yes	Yes	Yes	Yes	Yes	Yes	Yes	Yes	Yes	Yes	Yes	Yes	Yes	Yes	Yes	Yes	Yes	Yes	Yes	Yes	Yes	Yes
Passer domesticus	Yes	Yes	Yes	Yes	Yes	Yes	Yes	Yes	Yes	Yes	Yes	Yes	Yes	Yes	Yes	Yes	Yes	Yes	Yes	Yes	Yes	Yes	Yes	Yes		Yes	Yes	Yes
Passer montanus	Yes	Yes	Yes		Yes	Yes	Yes		Yes	Yes	Yes	Yes	Yes		Yes	Yes		Yes		Yes	Yes	Yes	Yes	Yes	Yes	Yes	Yes	Yes
Pica pica	Yes	Yes	Yes	Yes	Yes	Yes	Yes	Yes	Yes	Yes	Yes	Yes	Yes	Yes	Yes	Yes	Yes	Yes	Yes	Yes	Yes	Yes	Yes	Yes	Yes	Yes	Yes	Yes
Prunella modularis	Yes	Yes			Yes	Yes	Yes	Yes	Yes	Yes		Yes	Yes	Yes	Yes	Yes	Yes	Yes	Yes	Yes			Yes		Yes	Yes	Yes	Yes
Streptopelia decaocto	Yes	Yes	Yes	Yes	Yes	Yes			Yes	Yes	Yes	Yes	Yes	Yes				Yes		Yes	Yes		Yes	Yes	Yes	Yes	Yes	Yes
Sturnus vulgaris	Yes	Yes	Yes		Yes	Yes	Yes	Yes	Yes	Yes	Yes	Yes	Yes	Yes	Yes	Yes	Yes	Yes	Yes	Yes		Yes	Yes	Yes	Yes	Yes	Yes	Yes
Turdus merula	Yes	Yes	Yes		Yes	Yes	Yes	Yes	Yes	Yes	Yes	Yes	Yes	Yes	Yes	Yes	Yes	Yes	Yes	Yes	Yes	Yes	Yes	Yes	Yes	Yes	Yes	Yes

Table S8: 22 species selected for the computation of the urban multispecies index for each county.

Species names	Austria	Belgium	Bulgaria	Cyprus	Czech Republic	Denmark	Estonia	Finland	France	Germany	Greece	Hungary	Italy	Ireland	Latvia	Lithuania	Luxembourg	Netherlands	Norway	Poland	Portugal	Romania	Slovakia	Slovenia	Spain	Sweden	Switzerland	UK
Acrocephalus scirpaceus					Yes	Yes		Yes	Yes			Yes	Yes					Yes		Yes	Yes				Yes	Yes	Yes	Yes
Aegithalos caudatus	Yes				Yes	Yes	Yes	Yes	Yes			Yes	Yes	Yes	Yes			Yes		Yes	Yes		Yes		Yes	Yes	Yes	Yes
Alcedo atthis					Yes				Yes				Yes					Yes		Yes	Yes				Yes			Yes
Ardea cinerea	Yes		Yes			Yes			Yes			Yes	Yes	Yes		Yes		Yes	Yes	Yes	Yes			Yes	Yes	Yes	Yes	Yes
Buteo buteo	Yes		Yes		Yes	Yes	Yes		Yes		Yes	Yes	Yes		Yes	Yes	Yes	Yes		Yes	Yes		Yes	Yes	Yes	Yes	Yes	Yes
Carduelis carduelis	Yes		Yes	Yes	Yes	Yes	Yes		Yes		Yes	Yes	Yes	Yes	Yes	Yes		Yes		Yes	Yes	Yes	Yes	Yes	Yes	Yes	Yes	Yes
Chloris chloris	Yes		Yes	Yes	Yes	Yes	Yes	Yes	Yes		Yes	Yes	Yes	Yes	Yes	Yes	Yes	Yes	Yes	Yes	Yes		Yes	Yes	Yes	Yes	Yes	Yes
Ciconia ciconia			Yes		Yes				Yes		Yes	Yes			Yes	Yes		Yes		Yes	Yes		Yes		Yes		Yes	
Circus aeruginosus	Yes				Yes	Yes			Yes			Yes	Yes		Yes	Yes		Yes		Yes					Yes	Yes		
Coccothrauste coccothraustes	Yes		Yes		Yes	Yes	Yes		Yes			Yes	Yes		Yes	Yes	Yes	Yes		Yes	Yes		Yes	Yes	Yes	Yes	Yes	
Corvus frugilegus					Yes	Yes			Yes			Yes		Yes				Yes		Yes			Yes			Yes	Yes	Yes
Coloeus monedula	Yes		Yes	Yes	Yes	Yes	Yes	Yes	Yes		Yes		Yes	Yes	Yes	Yes	Yes	Yes		Yes			Yes	Yes	Yes	Yes	Yes	Yes
Cyanistes caeruleus	Yes		Yes		Yes	Yes	Yes	Yes	Yes		Yes	Yes	Yes	Yes	Yes	Yes	Yes	Yes	Yes	Yes	Yes	Yes	Yes	Yes	Yes	Yes	Yes	Yes
Emberiza calandra	Yes		Yes	Yes	Yes	Yes			Yes		Yes	Yes	Yes					Yes		Yes	Yes	Yes	Yes	Yes	Yes			Yes
Emberiza hortulana			Yes					Yes	Yes											Yes					Yes	Yes		
Fulica atra	Yes				Yes	Yes			Yes			Yes	Yes					Yes		Yes	Yes					Yes	Yes	Yes
Gallinula chloropus					Yes	Yes			Yes				Yes	Yes				Yes			Yes				Yes		Yes	Yes
Garrulus glandarius	Yes		Yes		Yes	Yes	Yes	Yes	Yes		Yes	Yes	Yes		Yes	Yes	Yes	Yes	Yes	Yes	Yes	Yes	Yes	Yes	Yes	Yes	Yes	Yes
Lanius collurio	Yes		Yes		Yes	Yes	Yes	Yes	Yes		Yes	Yes	Yes		Yes			Yes		Yes		Yes	Yes	Yes	Yes	Yes	Yes	
Linaria cannabina	Yes			Yes	Yes	Yes	Yes		Yes		Yes	Yes	Yes	Yes	Yes	Yes	Yes	Yes		Yes	Yes		Yes	Yes	Yes	Yes	Yes	Yes
Lullula arborea	Yes		Yes		Yes				Yes		Yes	Yes	Yes		Yes			Yes		Yes	Yes			Yes	Yes	Yes	Yes	
Luscinia megathynchos	Yes		Yes		Yes				Yes		Yes	Yes	Yes				Yes	Yes		Yes	Yes	Yes	Yes	Yes	Yes		Yes	Yes
Motacilla cinerea	Yes				Yes				Yes				Yes	Yes				Yes			Yes		Yes		Yes	Yes	Yes	Yes
Passer montanus	Yes		Yes		Yes	Yes	Yes		Yes		Yes	Yes	Yes		Yes	Yes		Yes		Yes	Yes	Yes	Yes	Yes	Yes	Yes	Yes	Yes
Perdix perdix	Yes				Yes	Yes			Yes			Yes						Yes		Yes								Yes
Phasianus colchicus	Yes		Yes		Yes	Yes		Yes	Yes			Yes	Yes	Yes				Yes		Yes		Yes	Yes	Yes	Yes	Yes		Yes
Picus viridis	Yes		Yes		Yes	Yes			Yes			Yes	Yes				Yes	Yes	Yes	Yes	Yes		Yes	Yes	Yes	Yes	Yes	Yes
Podiceps cristatus					Yes	Yes			Yes				Yes					Yes		Yes					Yes	Yes	Yes	Yes
Sitta europaea	Yes				Yes	Yes	Yes		Yes			Yes	Yes		Yes	Yes	Yes	Yes	Yes	Yes	Yes		Yes	Yes	Yes	Yes	Yes	Yes
Streptopelia decaocto	Yes		Yes	Yes	Yes	Yes			Yes		Yes	Yes	Yes	Yes				Yes		Yes	Yes		Yes	Yes	Yes	Yes	Yes	Yes
Streptopelia turtur	Yes		Yes	Yes	Yes		Yes		Yes		Yes	Yes	Yes		Yes	Yes		Yes		Yes	Yes	Yes	Yes	Yes	Yes		Yes	Yes
Sylvia atricapilla	Yes		Yes		Yes	Yes	Yes	Yes	Yes			Yes	Yes	Yes	Yes	Yes	Yes	Yes	Yes	Yes	Yes	Yes	Yes	Yes	Yes	Yes	Yes	Yes
Sylvia communis	Yes		Yes		Yes	Yes	Yes	Yes	Yes		Yes	Yes	Yes	Yes	Yes	Yes		Yes	Yes	Yes		Yes	Yes	Yes	Yes	Yes	Yes	Yes
Tachybaptus ruficollis					Yes	Yes			Yes				Yes					Yes		Yes					Yes		Yes	Yes
Turdus merula	Yes		Yes		Yes	Yes	Yes	Yes	Yes		Yes	Yes	Yes	Yes	Yes	Yes	Yes	Yes	Yes	Yes	Yes	Yes	Yes	Yes	Yes	Yes	Yes	Yes

Table S9: 35 species selected for the computation of the hot-dweller multispecies index for each county.

Species names	Austria	Belgium	Bulgaria	Cyprus	Czech Republic	Denmark	Estonia	Finland	France	Germany	Greece	Hungary	Italy	Ireland	Latvia	Lithuania	Luxembourg	Netherlands	Norway	Poland	Portugal	Romania	Slovakia	Slovenia	Spain	Sweden	Switzerland	UK
Acanthis flammea	Yes				Yes	Yes		Yes		Yes			Yes	Yes				Yes	Yes							Yes	Yes	Yes
Actitis hypoleucos					Yes			Yes	Yes										Yes	Yes	Yes				Yes	Yes		Yes
Anthus pratensis		Yes			Yes	Yes	Yes	Yes	Yes	Yes				Yes	Yes	Yes		Yes	Yes	Yes						Yes	Yes	Yes
Anthus trivialis	Yes	Yes			Yes	Yes	Yes	Yes	Yes	Yes		Yes	Yes		Yes	Yes	Yes	Yes	Yes	Yes		Yes	Yes	Yes	Yes	Yes	Yes	Yes
Bonasa bonasia							Yes	Yes							Yes											Yes	Yes	
Calcarius lapponicus								Yes											Yes							Yes		
Carpodacus erythrinus					Yes		Yes	Yes							Yes					Yes			Yes			Yes		
Certhia familiaris	Yes	Yes			Yes	Yes	Yes	Yes	Yes	Yes			Yes	Yes	Yes					Yes			Yes			Yes	Yes	Yes
Dryocopus martius	Yes	Yes			Yes	Yes	Yes	Yes	Yes	Yes		Yes	Yes		Yes	Yes		Yes	Yes	Yes			Yes	Yes	Yes	Yes	Yes	
Emberiza rustica								Yes																		Yes		
Emberiza schoeniclus		Yes			Yes	Yes		Yes	Yes	Yes		Yes		Yes	Yes	Yes		Yes	Yes	Yes			Yes			Yes	Yes	Yes
Ficedula hypoleuca					Yes	Yes	Yes	Yes	Yes	Yes					Yes	Yes		Yes	Yes	Yes					Yes	Yes	Yes	Yes
Fringilla montifringilla								Yes											Yes							Yes		
Gallinago gallinago					Yes	Yes	Yes	Yes		Yes					Yes			Yes	Yes	Yes						Yes		Yes
Grus grus							Yes	Yes		Yes					Yes	Yes			Yes	Yes						Yes		
Haematopus ostralegus						Yes		Yes										Yes	Yes							Yes		Yes
Larus ridibundus						Yes			Yes	Yes								Yes							Yes	Yes	Yes	Yes
Luscinia svecica								Yes											Yes							Yes		
Lyrurus tetrix							Yes	Yes							Yes				Yes							Yes	Yes	
Nucifraga caryocatactes	Yes				Yes		Yes		Yes				Yes		Yes											Yes	Yes	
Numenius arquata						Yes	Yes	Yes	Yes	Yes								Yes	Yes							Yes		Yes
Phylloscopus trochilus	Yes	Yes			Yes	Yes	Yes	Yes	Yes	Yes		Yes		Yes	Yes	Yes	Yes	Yes	Yes	Yes			Yes			Yes	Yes	Yes
Pluvialis apricaria								Yes											Yes							Yes		
Poecile montanus	Yes	Yes			Yes		Yes	Yes	Yes	Yes			Yes		Yes			Yes	Yes	Yes			Yes			Yes	Yes	Yes
Prunella modularis	Yes	Yes			Yes	Yes	Yes	Yes	Yes	Yes		Yes	Yes	Yes	Yes	Yes	Yes	Yes	Yes	Yes			Yes		Yes	Yes	Yes	Yes
Pyrrhula pyrrhula	Yes	Yes			Yes	Yes	Yes	Yes	Yes	Yes			Yes	Yes	Yes			Yes	Yes	Yes			Yes		Yes	Yes	Yes	Yes
Regulus regulus	Yes	Yes			Yes	Yes	Yes	Yes	Yes	Yes			Yes	Yes	Yes		Yes	Yes	Yes	Yes			Yes		Yes	Yes	Yes	Yes
Saxicola rubetra	Yes				Yes	Yes	Yes	Yes	Yes	Yes		Yes	Yes		Yes	Yes		Yes	Yes	Yes		Yes	Yes	Yes	Yes	Yes	Yes	Yes
Spinus spinus	Yes				Yes	Yes	Yes	Yes		Yes			Yes		Yes			Yes	Yes	Yes			Yes			Yes	Yes	Yes
Tringa glareola								Yes											Yes							Yes		
Tringa nebularia								Yes											Yes							Yes		
Tringa ochropus							Yes	Yes							Yes				Yes							Yes		
Tringa totanus						Yes		Yes	Yes	Yes		Yes						Yes	Yes	Yes						Yes		Yes
Turdus iliacus							Yes	Yes							Yes				Yes							Yes		
Turdus pilaris	Yes	Yes			Yes	Yes	Yes	Yes	Yes	Yes			Yes		Yes	Yes			Yes	Yes			Yes			Yes	Yes	

Table S10: 35 species selected for the computation of the cold-dweller multispecies index for each county.

Supplementary Information Appendix 6

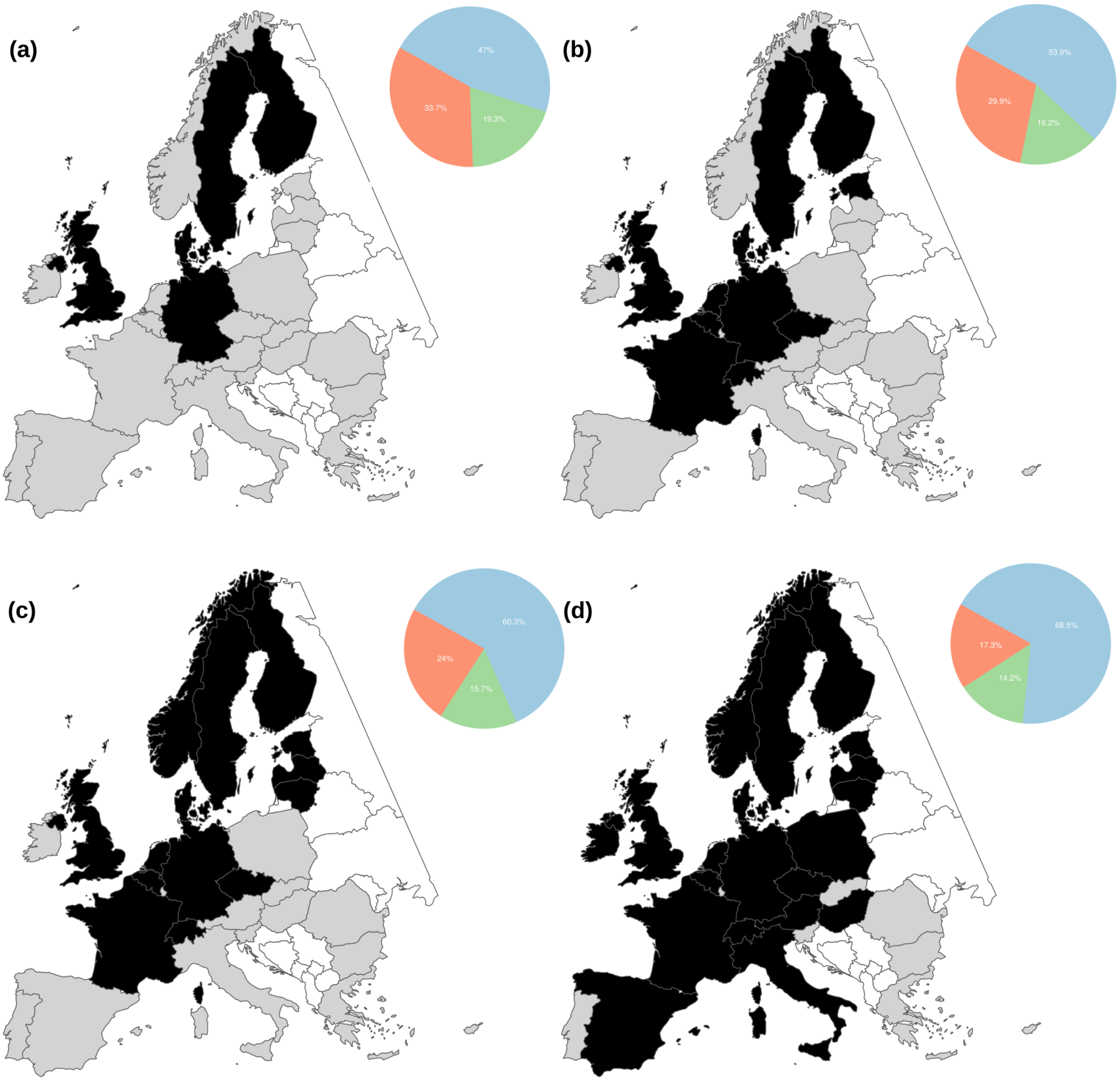

Figure S6: Impact of time period on country distribution and species trends. a) Trends and countries, 1981-2016. b) Trends and countries, 1991-2016. c) Trends and countries, 1996-2016 . d) Trends and countries, 2001-2016. Countries in grey were involved in the PanEuropean Common Bird Monitoring Scheme (PECBMS) in 2016 and in black countries in PECBMS during the period considered. Pie charts show the overall distribution of species trend between increasing (green), decreasing (orange) and non-significant (blue) trends. Overall, the best compromise between spatial coverage and temporal variability

(as few non-significant trends as possible) is between 1996 and 2016 with 39.7 % of significantly non-stable trends (24 % decreasing and 15.7% increasing).

Supplementary Information Appendix 7

Overall, we found a majority of unidirectional forcings from pressures to species, but bidirectional forcings between species and pressures and forcing from species to pressure have also been found (Tab. S10). Influence of species on pressures are false positive. In bidirectional forcing, a significant influence of species on pressures might be due to a strong synchrony between species and pressure time-series (3). One-way influence of species on pressures (i.e. when no forcing is found from pressure to species) are due to data limitation [(4)]. Acknowledging the limits of the data used in this study, we used two different tests to assess the quality and consistency of our approach: we tested the influence of removing each country in the detection of pressure influence and we used a test of false negative and false positive detection rate when the proportion and magnitude of influence between time-series change.

Pressure	Pressure -> Species	Pressure <-> Species	Pressure <- Species	No forcing
High-input farm cover	33 (8 farmland species)	18 (2 farmland species)	31	86
Forest cover	30 (3 forest species)	1 (1 forest species)	5	132
Urbanisation	21 (3 urban species)	8 (4 urban species)	17	122
Temperature	50 (10 hot dweller positively influenced, 4 hot dweller negatively influenced, 4 cold dwellers negatively influenced, 1 cold dweller positively influenced)	8 (1 hot dwellers positively influenced, 1 hot dweller negatively influenced, 1 cold dweller negatively influenced)	13	97

Table S11: Number of species affected by pressure (Pressure -> Species), bidirectional forcing (Pressure <-> Species), species affecting pressure (Pressure <- Species) and non forcing for each pressure.

1. One-by-one country removal test

We repeated the estimation of each pressure influence on each species while successively removing each country. Each time, we therefore used species and pressure time-series for the 27 other countries to which we applied the framework described in the main manuscript. We then analysed whether removing a given country impacted the number of species affected by a pressure (Fig. S6).

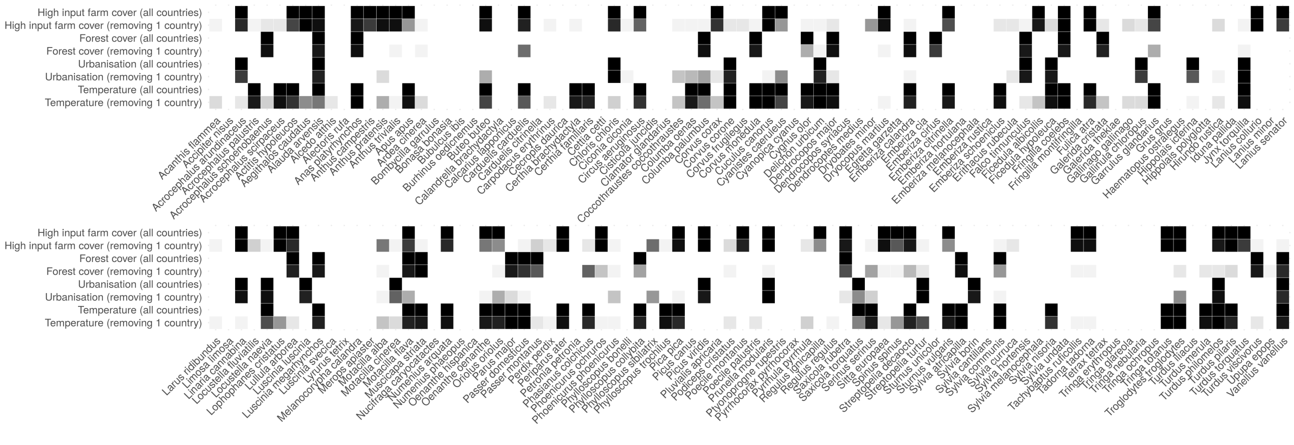

Figure S7: Influence of country removal in detecting pressure influence on species. For each pressure except for temperature (due to computational time), the influence calculated using time-series from all countries (*all countries*, black square when the influence is significant, white otherwise) are compared to the average influence obtained when successively removing each country (*removing 1 country*, grey scale proportional to the number of times the species was found influenced when removing successively each country). For instance, the Coal tit (*Periparus ater*) is affected by temperature when data from all countries are used (black square in the seventh row) and this remains valid when calculated on data in which each country is successively removed (black square in the eighth row). Conversely, the common redpoll (*Acanthis flammea*) is not affected by temperature when data from all countries are used (white square in the seventh row) but can be detected as being affected by temperature when some countries are successively removed (grey square in the eighth row).

Pressure	Affected with all countries or when removing one country.	Not significantly affected, neither with all countries nor when removing one country.	Not significantly affected with all countries but significantly affected when removing one country (at least for one of the countries).
High-input farm cover	50	69	49
Forest cover	25	117	26
Urbanisation	21	123	26
Temperature	55	73	40

Table S12: Number of species affected by each pressure when using all countries or when removing successively each country. Species found significantly affected when using all countries were also found significantly affected when removing successively each country. Most of the species not significantly affected when using all countries were not significantly affected when removing a given country. Some species not significantly affected when using all countries were found significantly affected when removing a given country.

Considering the results from a country-based perspective, we found three cases: countries without which fewer species are found significantly affected by the pressure (case 1), countries without which more species are found significantly affected by the pressure (case 2) and countries without which the number of species significantly affected by the pressure stayed the same (case 3). Removing time-series from a given country can change the estimation of the pressure influence by affecting the length of the time-series used and by increasing or decreasing the proportion of time-series with information. For instance, if a country where the influence of the considered pressure is high is removed, detecting the influence from the reduced dataset will be more difficult (see case 1). Conversely, the removal of time-series from a country where there is no or a weak influence of the pressure on the species may increase the proportion of time-series where the pressure influence is visible and therefore it will make it easier to detect the pressure influence (see case 2). Finally, if the removed country was not more or less informative than the average, its removal may not impact the final estimate (case 3).

Considering the results from a species-based perspective, we also found three cases (Tab. S11 and Fig. S6). Case 1: all species significantly affected when using all countries were also found to be influenced by pressure when removing a given country. Case 2: most of the species not significantly affected by the considered pressure were never found affected when removing a country. Case 3: some of the species not significantly affected when using all countries were found to be influenced when removing a given country. Cases 1 and 2 were expected and depict the consistency of our results when removing countries one-by-one. Case 3 corresponds to false negatives where the information on the pressure influence on the species seems to be present in the dataset but is diluted by data from some countries. Overall, our approach is conservative as we focused on pressure influences on species that are strong enough to not be diluted when considering all countries and shared between enough countries to not disappear when some countries are removed.

2. Testing the effect of pressure influence variability on the false negative and false positive rates.

We analysed the false positive and false negative rates of our method for each pressure influence. Initially, the multispatial CCM method had been tested on time-series simulated with an interaction strength constant across plots and on an empirical example[53] was based on analysing plant-plant competition in spatially close plots and thus the interaction strength was not expected to differ substantially between plots. In our study, each of the pressures analysed could have a different influence on the different populations of a given species as our spatial replicates are countries (*i.e.* spatial replicates are not spatially close). We therefore tested the effect of variability in pressure influence between countries on the false negative and false positive rates. Hence, we analysed the average false positive and false negative rates by using simulated data which mimic species-pressure datasets (long pseudo-time-series of one species and one pressure reconstructed from national time-series). To do so, we simulated data using the R function *make_ccm_data* from the R package *multispatialCCM* [(4)]. This provides a simulated dataset with two time-series from a two species competition model where species *B* negatively affect species *A* but species *A* does not affect species *B*. In our case, *B* corresponds to the pressure and *A*

to the species. This function admits several parameters to model the simulated dataset as close as possible to the observed species-pressure dataset. The *number of sequential observations* corresponded to the average times-series length by country and has been set to 10. The *total number of time-series* corresponded to the average number of countries and has been set to 28. Hereafter we focused on a third parameter which is the *forcing strength* between *B* and *A*.

We tested the effect of the variability in the *forcing strength* (i.e. pressure influence) among countries in three steps.

First, we tested the effect of the proportion of time-series without forcing strength on the false negative and false positive detection rates. We used six different proportions of time-series with a forcing strength set to 0, evenly spaced from 0 to 100 % (0 %, 20 % , 40 % , 60 %, 80 %, 100 %). There are two kinds of false positive rates. The first one corresponds to the number of times a significant effect was found from *B* to *A* although there was no effect (100 % of time-series with a forcing strength set to 0) among the number of trials. The second one corresponds to the number of times a significant effect was found from *A* to *B*, although *A* is not supposed to influence *B*. False negative rates correspond to the number of times no significant effect was found between *B* and *A* although there was an effect (between 20 % and 100 % of time-series with a forcing strength set to 1.25 (4)) among the number of trials.

In a second step, we tested the effects of the variability in the forcing strength by drawing values from five different intervals centred on 1.25 ([1.2, 1.3], [0.9, 1.6], [0.6, 1.9], [0.3, 2.2], [0, 2.5]).

Finally in a third step, we tested the effect of the intensity if forcing strength on its detectability. We used a forcing strength different from 0 for only one country among the 28 used to build the long pseudo-time-series. For that country, we tested five different values of the forcing strength, evenly spaced from 0.3 to 2.5 (0.30, 0.85, 1.40, 1.95, 2.50). Forcing strengths in all other countries were set to 0.

Each of these three tests was conducted 1000 times. This results in 16,000 ((6 + 5 + 5) x 1000) combinations to which was applied the multispatial CCM with 100 bootstrap iterations (due to computing time[53]) corresponding to a total of 1.6 million individual multispatial tests.

Percentage of countries with forcing strength set to 0	0 %	20 %	40 %	60 %	80 %	100 %
False negative rate of significant effect from B to A (except from the last column: false positive rate).	0.006	0.311	0.375	0.560	0.796	0.119

Table S13: Average false negative and false positive rates of significant effect from B to A for each proportion of time-series with forcing strength set to 0.

Forcing strength in	[1.2, 1.3]	[0.9, 1.6]	[0.6, 1.9]	[0.3, 2.2]	[0, 2.5]
False negative rate of significant effect from B to A	0.239	0.264	0.502	0.493	0.323

Table S14: Average false negative rates of significant effect from B to A for each interval of forcing strength.

Forcing strength (different from 0 in only one country)	0.30	0.85	1.40	1.95	2.5
False negative rate of significant effect from B to A	0.881	0.741	0.836	0.866	0.980

Table S15: Average false negative rates of significant effect from B to A for each forcing strength (different from 0 in only one country).

High false negative rates and low false positive rates (the average false positive rate of significant effect from *A* to *B* is 0.085) were consistent with previous results [(4)]. False negative rates decrease when the proportion of time-series with a non null influence of pressure increases (Tab. S12). False negative rates increase with the variability in pressure influence among countries (Tab. S13). When only one time-series has a non-null strength the strength value does not affect the detection rate which remains very low (Tab. S14). All together these results show that the approach used tends to detect only existing influence (low false positive rates, Tab. S12) but fails to detect existing influence when time-series are short or when strength variability is high. This may explain the low number

of species affected by urbanisation or forest cover. Moreover the higher number of species affected by high-input farm cover than by temperature although time-series were shorter for the first pressure may be the result of a stronger variability in the influence strength of temperature compared to a more stable influence strength of high-input farm cover.

Supplementary Information Appendix 8

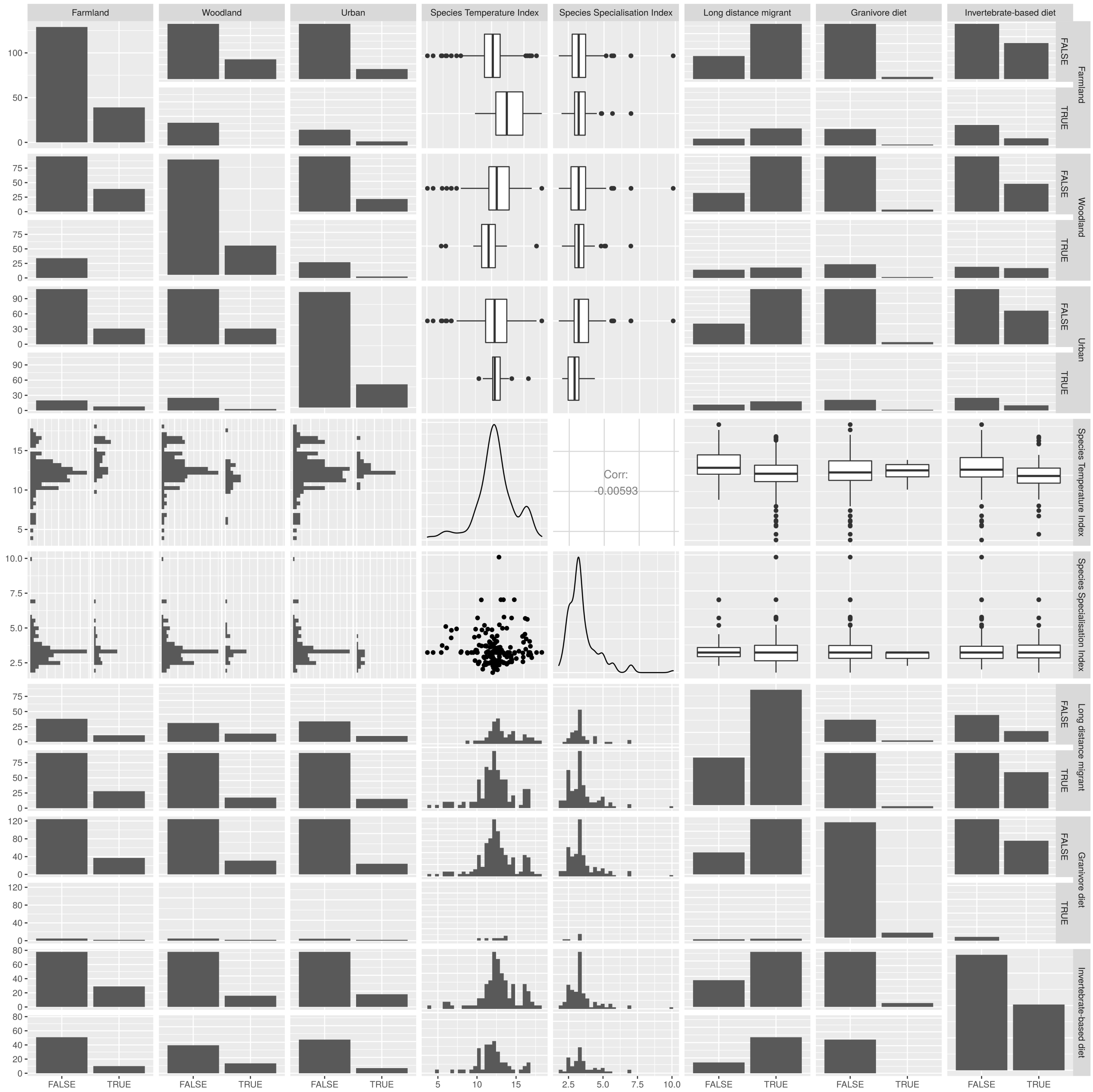

Figure S8: Density plots and correlations between species traits. Each dot represents a species.

SI references:

1. R. Inger, *et al.*, Common European birds are declining rapidly while less abundant species' numbers are rising. *Ecol. Lett.* **18**, 28–36 (2015).

2. EEA, Farm structure, European Environment Agency, available at https://ec.europa.eu/eurostat/fr/data/database and see metadata for detail at https://ec.europa.eu/eurostat/cache/metadata/en/ef_sims.htm. (2020).

3. G. Sugihara, *et al.*, Detecting causality in complex ecosystems. *Science* **338**, 496–500 (2012).

4. A. T. Clark, *et al.*, Spatial convergent cross mapping to detect causal relationships from short time series. *Ecology* **96**, 1174–1181 (2015).
